# Enabling laboratory readiness and preparedness for the evaluation of suspected viral hemorrhagic fevers: development of a laboratory toolkit

**DOI:** 10.1017/ice.2024.143

**Published:** 2024-09

**Authors:** Sarah E. Turbett, Jacob E. Lazarus, Mia A. Nardini, Joseph E. Braidt, Stefanie A. Lane, Eileen F. Searle, Paul D. Biddinger, Erica S. Shenoy

**Affiliations:** 1 Division of Infectious Diseases, Massachusetts General Hospital, Boston, MA, USA; 2 Department of Pathology, Massachusetts General Hospital, Boston, MA, USA; 3 Harvard Medical School, Boston, MA, USA; 4 Region 1 Regional Emerging Special Pathogens Treatment Center, Boston, MA, USA; 5 Center for Disaster Medicine, Massachusetts General Hospital, Boston, MA, USA; 6 Department of Emergency Medicine, Massachusetts General Hospital, Boston, MA, USA; 7 Infection Control Unit, Massachusetts General Hospital, Boston, MA, USA; 8 Infection Control, Mass General Brigham, Boston, MA, USA

## Abstract

**Purpose:**

Viral hemorrhagic fevers (VHFs), such as Ebola virus disease, Marburg virus disease, and Lassa fever, are associated with significant morbidity and mortality and the potential for person-to-person transmission. While most individuals in whom VHF is suspected will ultimately be diagnosed with a non-VHF illness, such patients may present to any United States healthcare facility (HCF) for initial evaluation; therefore, all HCFs must be prepared to evaluate and initiate care for suspect VHF patients, especially if they are acutely ill. Included within this evaluation is the ability to perform basic routine laboratory testing before VHF-specific diagnostic test results are available, as well as rapid malaria testing to assess for a common, dangerous “VHF mimic.”

**Objective:**

To improve laboratory preparedness and readiness in the initial care of suspect VHF patients who may present to acute care hospitals.

**Design:**

Plan-Do-Study-Act quality improvement model.

**Setting:**

Frontline healthcare facilities and their clinical laboratories.

**Methods:**

We describe the development of a laboratory testing toolkit for a suspect VHF patient that can assist frontline HCFs in providing basic laboratory testing required for the care of these patients.

**Results:**

The toolkit provides guidance on infection prevention and control, waste management, occupational health, laboratory test collection, processing, and resulting, in the context of suspect VHF patient evaluation.

**Conclusions:**

The toolkit is designed to be readily adapted by any frontline HCF in the US. With the guidance provided, facilities will be able to support safer initial evaluation of VHF suspects and ensure high-quality patient care.

## Introduction

Viral hemorrhagic fevers (VHFs), such as Ebola virus disease, Marburg virus disease, and Lassa fever, are a group of emerging infections categorized as high-consequence infectious diseases due to a combination of clinical and epidemiological characteristics.^1^ Clinically, VHFs are characterized by their high levels of acuity, morbidity and mortality, person-to-person transmissibility, and also by a lack of readily available therapeutic countermeasures.^2^ Early identification and isolation of individuals presenting with signs and symptoms consistent with, and epidemiological risk factors for VHF is critical for diagnosis. It is also necessary to prevent nosocomial transmission to patients, visitors, and healthcare personnel.^3^

However, because VHF risk is low in non-endemic and non-outbreak settings, and the clinical signs and symptoms of VHF are often nonspecific, US healthcare facilities (HCFs) face challenges to rapidly identify and isolate suspect VHF patients. While very few HCFs will ever encounter a patient with a VHF as most patients initially evaluated for VHF will be found to have an alternative diagnosis, failure to identify a potential VHF can result in unintended exposure and transmission risk.

Though patients with a VHF can present to any US HCF, the number of US HCFs with established laboratory protocols to perform routine diagnostic testing (eg electrolytes) on samples from suspect VHF patients is unknown. Given the relative rarity of VHFs and the stringent laboratory safety protocols needed to minimize risk of workplace exposure, establishing and maintaining these laboratory practices is challenging for HCFs, likely limiting their implementation in most settings. Lack of access to routine laboratory testing may result in additional patient morbidity and mortality for multiple reasons. For example, for a patient with potential VHF, the inability to perform basic chemistries and electrolytes can delay important results necessary for supportive care. Additionally, being unable to rapidly diagnose more common “VHF mimics” such as malaria, can lead to delays in effective treatment that can significantly impact patient morbidity and mortality.^4^ In fact, the Centers for Disease Control and Prevention (CDC) has identified a delay in malaria diagnosis and treatment as the major contributor to fatal malaria cases in the US.^5^ As malaria is more common among febrile returning travelers than VHF, having access to rapid malaria diagnostics is crucial for optimal management of suspect VHF patients. Currently, only one rapid malaria diagnostic test (BinaxNOW Malaria, Abbott Diagnostics, Scarborough, Maine) is Food and Drug Administration-approved for use in clinical laboratories to aid in the diagnosis of malaria.^6^ It is estimated that fewer than 0.5% of clinical laboratories in the US perform rapid malaria testing based on 2023 College of American Pathologists survey data.^7,8^

Most recently, an outbreak of *Sudan ebolavirus* in the fall of 2022 exposed the challenges facing clinical laboratories related to frontline laboratory preparedness.^9^ To support the laboratory evaluation of travel-related Ebola virus disease cases for hospitals across the New England Region, planning began within the US Department of Health and Human Services (HHS) Region 1 Regional Emerging Special Pathogens Treatment Center (RESPTC) at the Massachusetts General Hospital (MGH). This planning included infection prevention and control, emergency preparedness and laboratory leaders at MGH, and collaboration with public health officials. During this planning, we identified gaps across the region in frontline facility laboratory preparedness.

As part of the RESPTC mission to support regional preparedness through evidence-based approaches and innovative tools and education, we conceptualized and developed a Laboratory Testing Toolkit for a suspect VHF Patient. The Toolkit, described here, is intended to provide generalizable and scalable resources to facilitate the development of local standard operating procedures for frontline clinical laboratories for the care of a suspect VHF patient.

## Methods

### Stakeholder identification and gap identification

In response to the outbreak of Ebola virus disease in Uganda in the fall of 2022 and Marburg virus disease in Equatorial Guinea in early 2023, HCF within the Mass General Brigham (MGB) system requested assistance to enhance their laboratory capabilities. These requests and inquiries from other HCF outside of MGB, coupled with the awareness of the rarity of a HCF laboratory to have rapid malaria testing, prompted Region 1 RESPTC leadership to prioritize the creation of this specialized Toolkit. MGB is a large integrated healthcare system in Massachusetts and New Hampshire and includes ten acute care facilities ranging from small facilities (hosting 14 licensed beds and 12,000 emergency department visits per year) to large facilities (hosting 1,045 licensed beds and 118,000 emergency department visits per year). For reference, the largest MGB facility has one of the 25 busiest emergency departments in the United States.^10^

In February and March of 2023, stakeholders in HHS Region 1 were identified. Key stakeholders included laboratory, infection prevention and control, and emergency preparedness experts at the state and a mix of academic medical centers, community, and critical access hospitals.

Between March 22, 2023, and May 15, 2023, the Massachusetts General Hospital RESPTC engaged with state health department officials from the six HHS Region 1 states. State health department officials were queried for additional state-level and HCF-level laboratorian leadership for further engagement. HCFs were assessed to have variable knowledge and resources required to develop and implement laboratory protocols for collection, processing, and analysis of specimens for initial laboratory testing of a suspect VHF patient. Additionally, HCFs required additional support for safe collection and expedited transport of VHF-specific confirmatory diagnostic specimens to state public health laboratories.

### Establishment of toolkit structure and content

To develop the Toolkit, a working group was convened comprising members from the Region 1 RESPTC, MGH microbiology laboratory, core laboratory, infection prevention and control, emergency medicine, occupational health, and infectious diseases services. Using the Clinical Laboratory Improvement Amendments (CLIA) quality standards for laboratory testing, public health guidance from CDC, and internal standard operating procedures and established resources resulting from the collaboration between the Region 1 RESPTC and the MGH clinical laboratory, the Toolkit was developed with two main components: a base plan that outlines facility-level planning of initial laboratory testing of a suspect VHF patient; and an appendix inclusive of adaptable resources such as checklists, templates, and other resources to facilitate local adoption. The development team placed specific focus on each component of the laboratory testing process (from specimen collection and lab test menu to test result reporting) to ensure that all elements of the testing procedure were addressed.^11^ Infection prevention and control considerations including personal protective equipment, cleaning and disinfection, and waste management were also incorporated into the document where applicable in accordance with guidance from CDC. All materials were structured to be applicable across a diverse set of acute care facilities and adaptable for individual facility use.

### Vetting curated content and application of plan-do-study-act cycles

To ensure that Toolkit content was relevant, current, reliable, and evidence-based, iterations of the Toolkit were vetted through subject matter experts using a Plan-Do-Study-Act framework. The plan phase is detailed above. The Study phase included collecting feedback from various end-user groups to assess the utility and completeness of the Toolkit. The Act phase was implemented in the winter of 2023–2024 and involved distribution of the Toolkit to acute care facilities within the MGB healthcare system.

## Results

### Subject matter expert and stakeholder engagement

The evolution of the Toolkit is detailed in Supplemental Figure 1. Over the eight-month period, twenty-four meetings or outreach events occurred to inform Toolkit development. The toolkit development team met six times to inform the Toolkit’s structure and content. Additionally, seven meetings with subject matter experts provided content-specific insight for resource development. To bolster the Toolkit’s applicability and relevance among diverse facilities, the team engaged in nine meetings with state health department officials from the six HHS Region 1 states.

### Toolkit structure: base plan

The Toolkit base plan consists of four sections designed to provide guidance through all phases of laboratory testing. The first section highlights key infection prevention and control, waste management, and occupational health considerations while the remaining three sections provide guidance on laboratory test collection, processing, and resulting. Laboratory testing is divided into pre-analytic, analytic, and post-analytic phases per CLIA guidance to ensure quality standards for testing are addressed during each phase of the testing process.^11^

### Infection prevention and control, waste management, and occupational health

**Figure 1.**
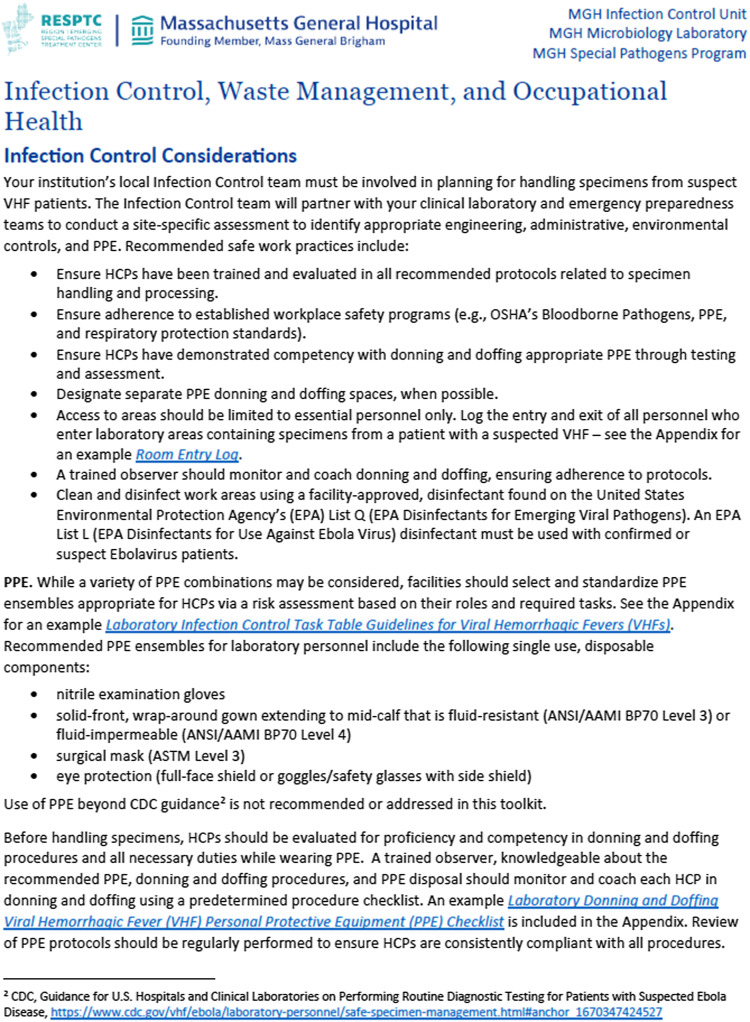
Recommended safe work practices and personal protective equipment for viral hemorrhagic fever suspect specimen handling and processing. HCP, health care personnel; OSHA, Occupational Health and Safety Administration; EPA, Environmental Protection Agency; ANSI, American National Standards Institute; AAMI, Association for Advancement of Medical Instrumentation; ASTM, American Society for Testing and Materials.

The infection prevention and control, waste management, and occupational health sections highlight important infection prevention and control considerations when performing laboratory testing for a suspect VHF patient. Included in this section are recommendations for selection, standardization, and training in the use of personal protective equipment (PPE) (Figure 1). Also discussed is the role of a trained observer competent in donning and doffing procedures and PPE disposal, who can coach health care personnels in proper technique. This section also references the cleaning and disinfection of equipment, waste management, and provides occupational health guidance in the setting of a potential VHF exposure.

### Pre-analytic considerations

**Figure 2.**
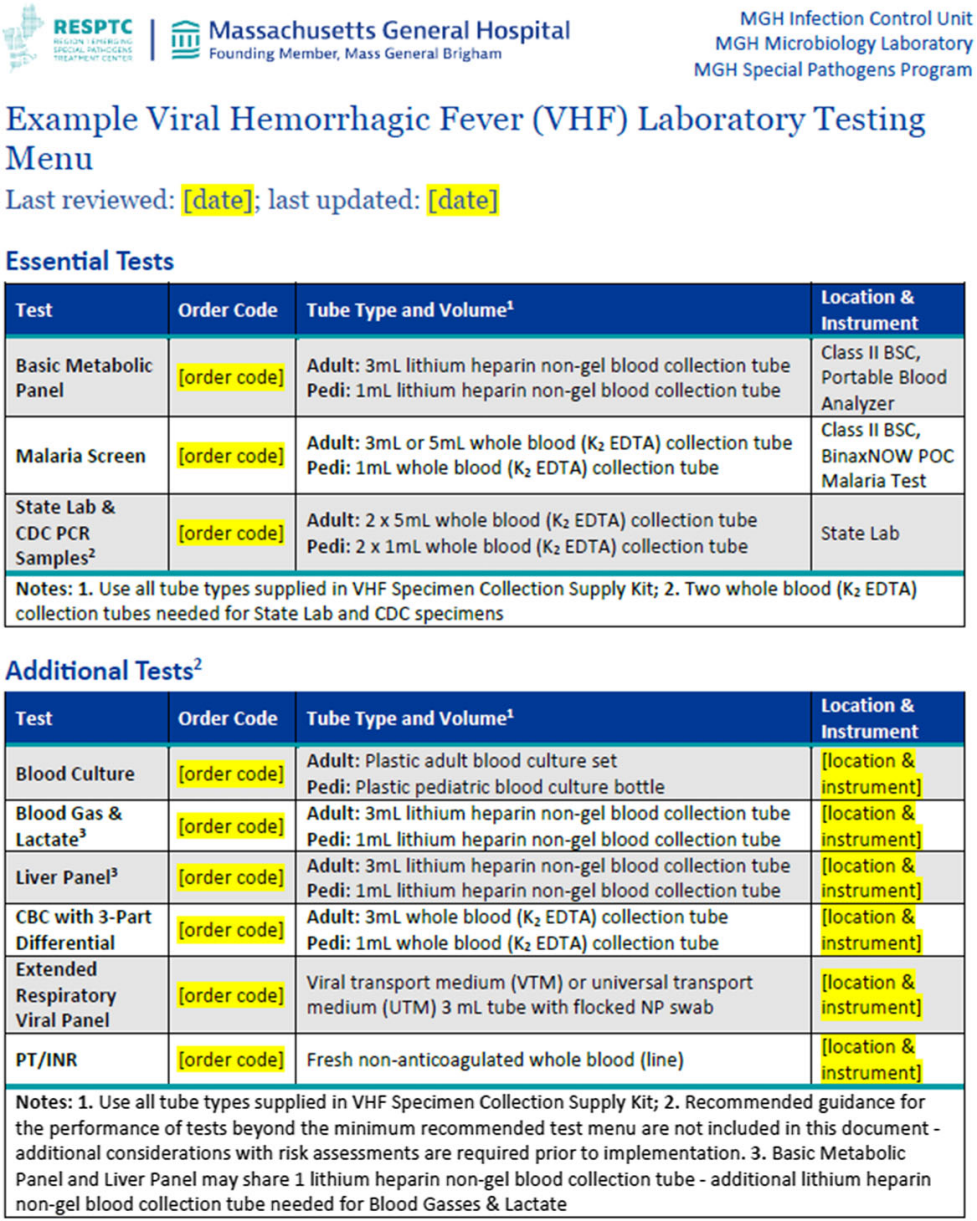
Example viral hemorrhagic fever laboratory testing menu. BSC, biosafety cabinet; EDTA, Ethylenediamine tetraacetic acid; POC, point of care; CDC, Centers for Disease Control and Prevention; PCR, polymerase chain reaction; CBC, complete blood count; VTM, viral transport medium; UTM, universal transport medium; NP, nasopharyngeal; PT/INR, prothrombin time/international normalized ratio.

The pre-analytic section describes best practices for laboratory test selection, specimen collection and transportation, laboratory accessioning, and specimen storage. For laboratory test selection, a lab menu outlining the minimum testing necessary for the evaluation and management of a suspect VHF patient is presented. Inherent to test selection is the recommendation that all laboratory testing be performed in the clinical laboratory space (as opposed to testing at or near the point of care) by laboratory professionals for improved adherence to testing procedures, maintenance of competency, and regulatory oversight. When determining the lab menu, the toolkit development team prioritized specific testing that can be rapidly and safely performed by the clinical laboratory. Included in this menu is basic chemistry and electrolyte testing to identify life-threatening electrolyte, renal, and acid-base abnormalities and rapid malaria testing to assess for a common alternative diagnosis in suspect VHF patients (Figure 2). For basic chemistry and electrolyte testing, handheld blood analyzers that can be operated within a Class II Biosafety Cabinet (BSC) are recommended. For rapid malaria testing, the commercially available BinaxNOW Malaria test provides a simple and accurate screening tool for *Plasmodium falciparum* infection with 96% sensitivity and 99% specificity compared to blood smear microscopy.^12^ Like handheld blood analyzers, it can be safely performed within a Class II BSC.^13^ Although blood smears for malaria microscopy are still required to confirm rapid malaria test results, due to laboratory safety considerations, these should not be performed in suspect or confirmed VHF patients, and treatment for malaria can be initiated prior to confirmatory testing.^14^

**Figure 3.**
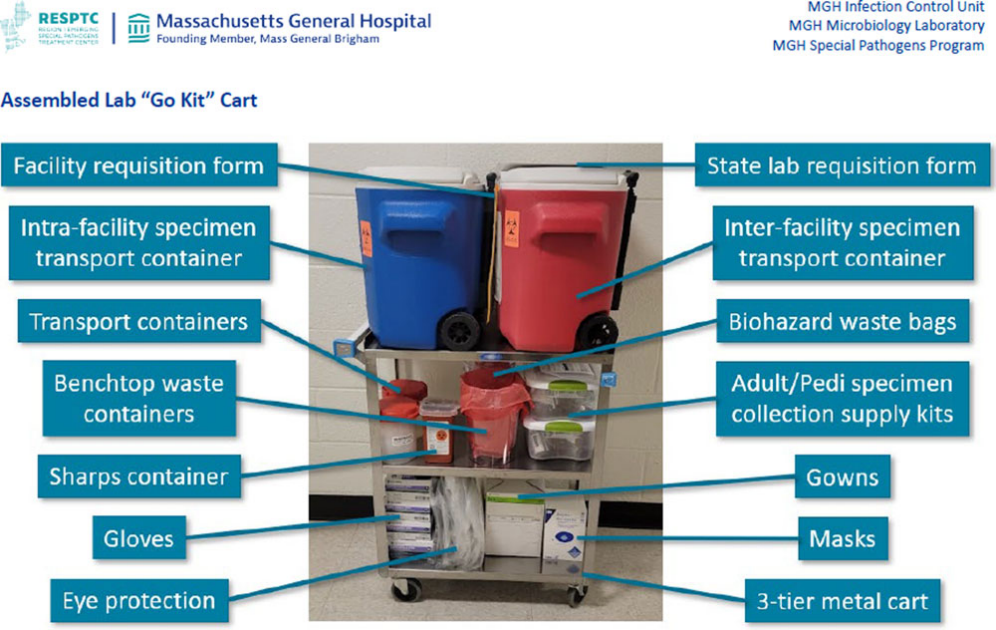
Assembled Laboratory “Go Kit” Cart.

An additional component of the pre-analytic section includes the description of a laboratory “go kit” cart which includes all necessary equipment and supplies to support laboratory testing of a suspect VHF patient (Figure 3). Self-contained specimen collection supply kits are also recommended to ensure appropriate specimen collection.

### Analytic considerations

**Figure 4.**
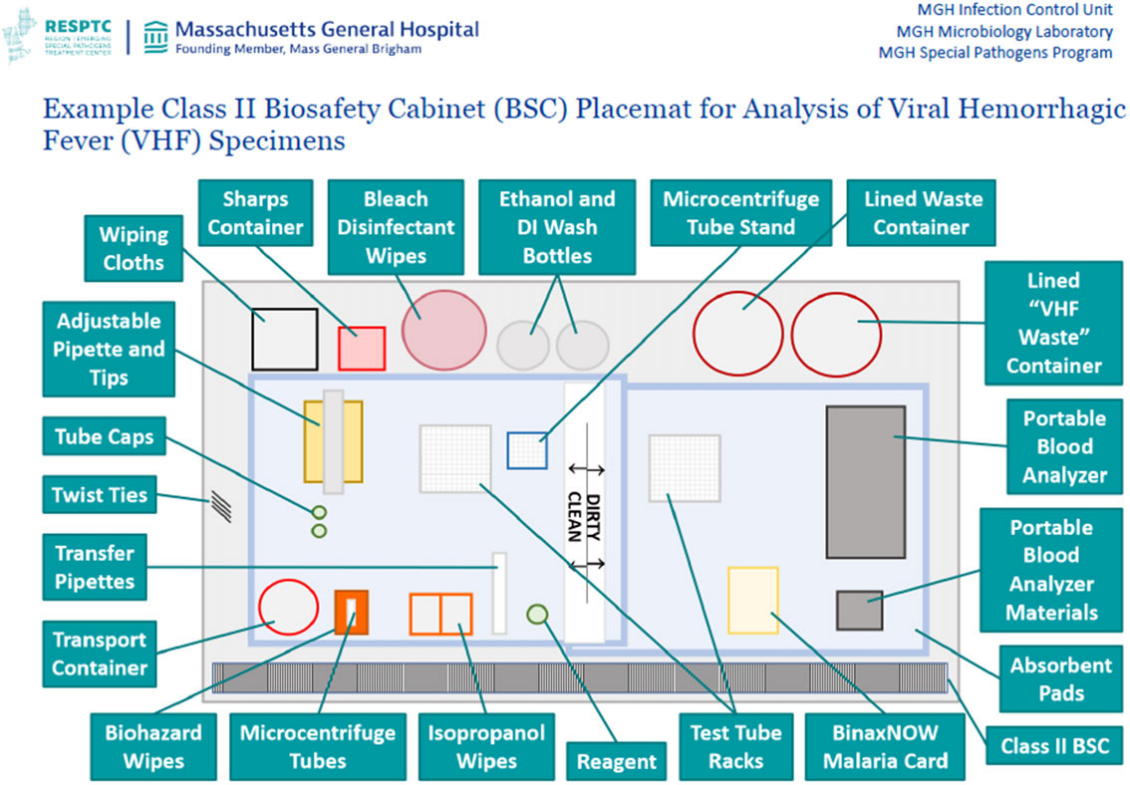
Picture map of class II biosafety cabinet placement for analysis of viral hemorrhagic fever specimens.

The analytic section provides guidance on preparing a safe workspace, safe specimen manipulation and processing, and laboratory test result reporting. This section includes recommendations for laboratory staffing assignments, which involve the assignment of two trained technologists for specimen processing. One technologist serves as the “testing tech” and is responsible for completing the testing within the Class II BSC. The other technologist serves as the “buddy tech” and is responsible for laboratory testing components occurring outside of the Class II BSC. It also includes instructions for BSC preparation, with the intent of creating well-differentiated “clean” and “dirty” areas within the BSC (Figure 4).

### Post-analytic considerations

The post-analytic section covers considerations specific to transporting VHF specimens from the facility to the public health laboratory. Specific guidance on safe specimen packaging and shipping is also provided.

### Toolkit structure: appendix

The Toolkit Appendix is formatted to include example documents and templates that clinical laboratories can adapt for local use. Additional checklists and task sheets are intended to further support adoption and implementation at HCFs that have previously not enacted Class II BSC laboratory testing. Packing lists for a laboratory “go kit” and specimen collection supply kit, as well as sample documents including a clinical lab requisition and test result form are provided. Example checklists for laboratory activation, pre-analytical specimen receipt and accessioning, sample processing and resulting, and Category A waste handling are included. An infection prevention and control task table describing PPE recommendations based on task, and donning and doffing checklists are included.

## Discussion

VHFs are an emerging group of infections of high clinical consequence due to their significant morbidity and mortality and their potential transmission without prompt initiation of appropriate infection prevention and control measures. The identification and management of suspect VHF patients remains challenging for HCFs, due to a paucity of frontline facility experience and familiarity with protocols for routine laboratory testing on samples from patients with suspected VHFs. Here, we describe the development of the Laboratory Testing Toolkit intended as a resource for HCF laboratories to support initial laboratory testing for a suspect VHF patient.

This step-by-step guide outlines best laboratory practices throughout all stages of the testing process and is expected to reduce the burden placed on frontline HCF to independently develop processes and procedures. By providing templated checklists and other documents that can be adopted and modified for local settings, clinical laboratories can ensure safe laboratory practices are followed while providing critical test results to clinicians in a timely manner. In addition to ensuring that frontline clinicians have access to these critical test results, this Toolkit also emphasizes less visible, yet crucial laboratory capabilities, including expedient packing and shipping of Category A samples, which are necessary to minimize the time from sample collection to sample result.

Certain tradeoffs are necessary when designing readiness and preparedness measures for suspect VHF patients. This Toolkit details the minimum laboratory testing necessary for safe supportive care of the VHF suspect prior to ruling out VHF disease or, alternatively, transfer to a higher level of care. Although CDC currently recommends performing numerous clinical laboratory tests in the evaluation of a suspect VHF patient, most frontline clinical laboratories do not have the infrastructure or resources available to provide the entire recommended menu. In addition to the tests in the Toolkit, the CDC recommends immediate blood smears with same-day results for malaria testing, complete blood count with differential, liver function tests, coagulation testing, urinalysis, and blood cultures.^15^ Of the tests recommended by CDC, this Toolkit focuses only on the basic metabolic panel and rapid malaria testing as essential procedures for frontline laboratories since severe electrolyte derangements can be rapidly fatal, as can untreated falciparum malaria. Should facilities have the capacity for expanded laboratory testing, the Toolkit is designed to be adaptable, with resources available to guide additional testing.

Although the Toolkit is designed to facilitate initial laboratory testing of a VHF suspect, there are limitations that frontline clinical laboratories must consider. First, although rapid malaria testing is available, most clinical laboratories do not routinely perform this testing due to the overall low prevalence of malaria in the US^16^ Standard laboratory practice requires test verification to confirm performance characteristics established by the manufacturer prior to reporting patient results.^17^ Verification is often accomplished by testing well-characterized clinical specimens which, for malaria, will be challenging for many clinical laboratories given the paucity of clinical cases in the US. Second, although the Toolkit does provides resources for many aspects of the laboratory testing process, it does not provide examples of verification protocols, laboratory test training, proficiency testing, or competency assessment; individual laboratories will need established procedures for these items prior to patient testing and reporting. Finally, although this Toolkit is derived from recommendations from CDC, guidance from local and state health departments supersedes any included information; state and local health departments must be contacted for advice should a VHF suspect present to a frontline facility.^17^

In summary, the Laboratory Testing Toolkit developed and described here should enable wider implementation of safe and expedited testing of suspect VHF patients. This framework has the potential to improve readiness and preparedness in anticipation of travel-related VHF cases.

## Supporting information

Turbett et al. supplementary material 1 Turbett et al. supplementary material

Turbett et al. supplementary material 2 Turbett et al. supplementary material

Turbett et al. supplementary material 3 Turbett et al. supplementary material

## Supplementary material

The supplementary material for this article can be found at https://doi.org/10.1017/ice.2024.143
