# Supplementary figures and images for: Enabling laboratory readiness and preparedness for the evaluation of suspected viral hemorrhagic fevers: development of a laboratory toolkit
Source: Infect Control Hosp Epidemiol. 2024 Oct 11;45(9):1043–9. doi: 10.1017/ice.2024.143 (PMC11518664; doi:10.1017/ice.2024.143)

Supplemental Figure 1. Timeline of Toolkit Development


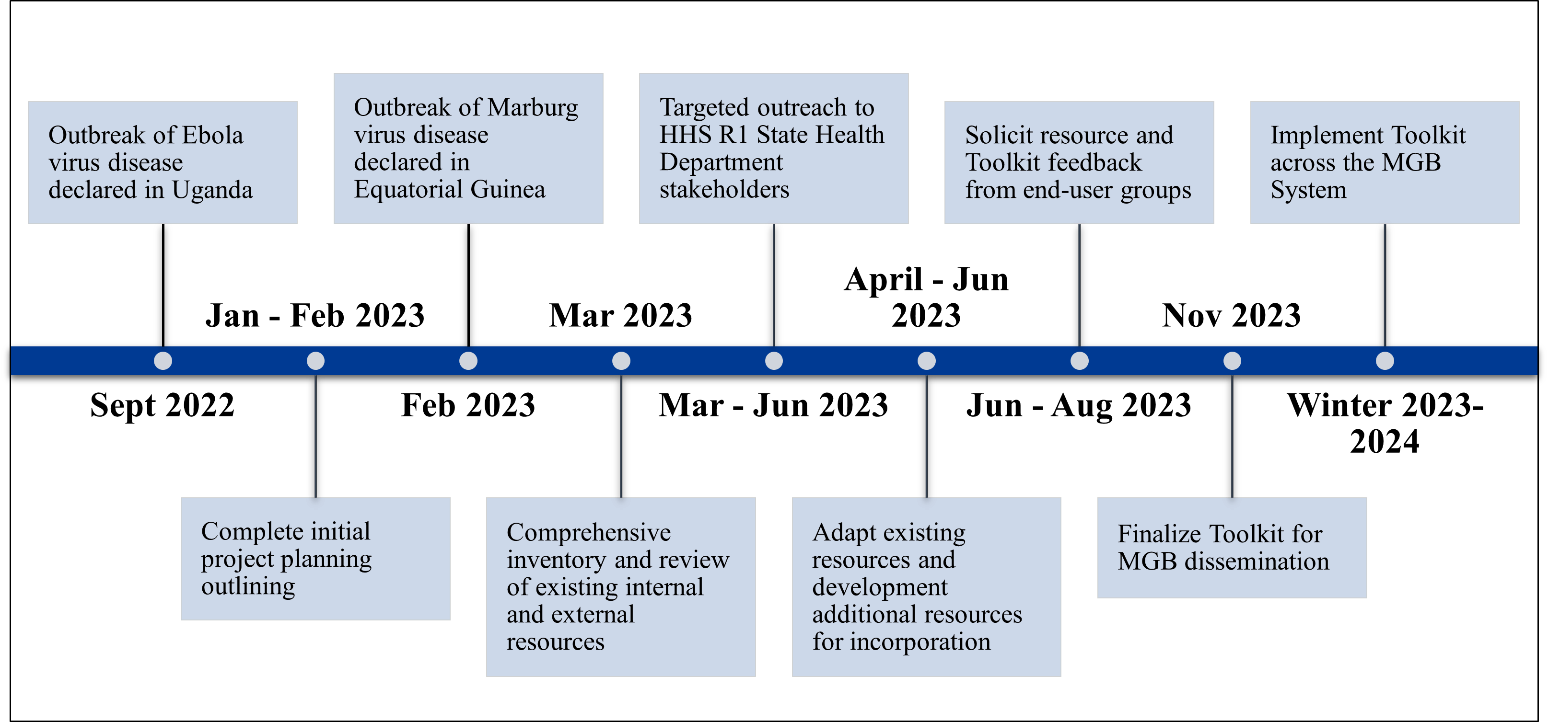

Supplement: Turbett et al. supplementary material 1 — Turbett et al. supplementary material [file S0899823X24001430sup001.docx]
