# Supplementary material for: Enabling laboratory readiness and preparedness for the evaluation of suspected viral hemorrhagic fevers: development of a laboratory toolkit
Source: Infect Control Hosp Epidemiol. 2024 Oct 11;45(9):1043–9. doi: 10.1017/ice.2024.143 (PMC11518664; doi:10.1017/ice.2024.143)
Supplement: Turbett et al. supplementary material 2 — Turbett et al. supplementary material [file S0899823X24001430sup002.docx]

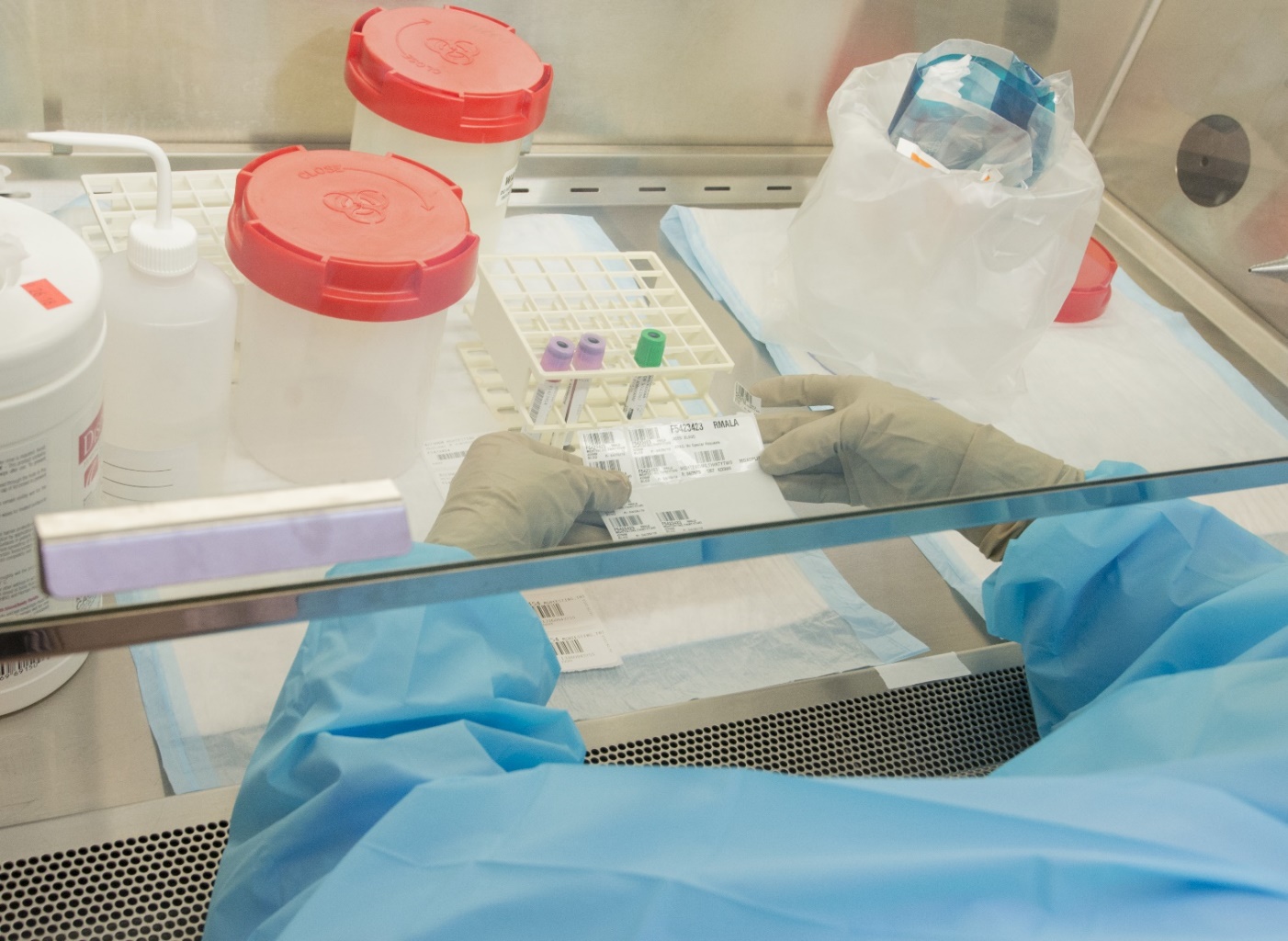

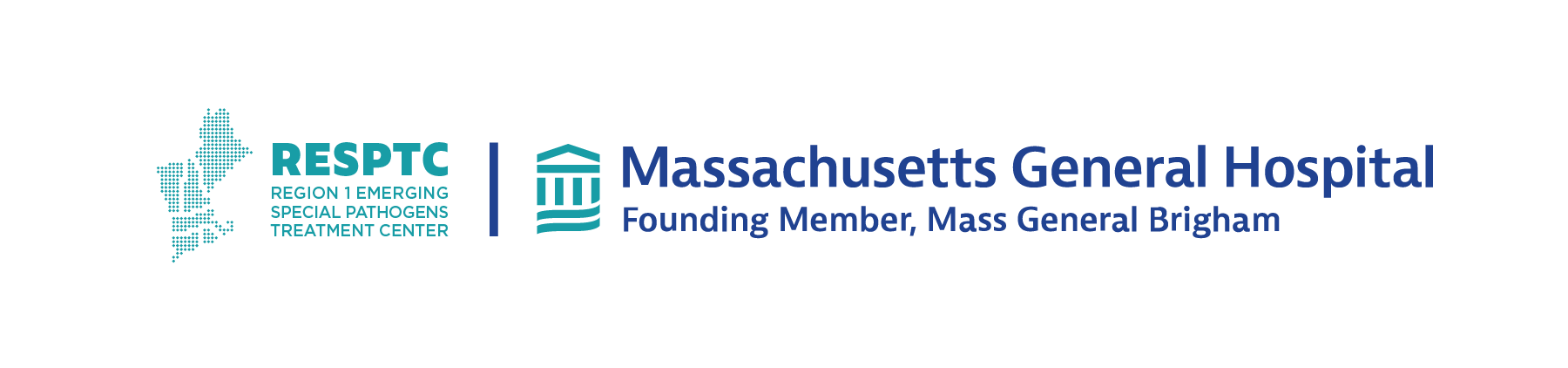


Laboratory Testing Toolkit for a Suspect Viral Hemorrhagic Fever (VHF) Patient

Example Documents and Templates Appendix

*November 2023*

**Table of Contents**

Introduction 3

Example Viral Hemorrhagic Fever (VHF) Laboratory Testing Menu 4

Example Viral Hemorrhagic Fever (VHF) Laboratory “Go Kit” Cart Packing List 5

Example Viral Hemorrhagic Fever (VHF) Specimen Collection Supply Kit Packing List 8

Example Viral Hemorrhagic Fever (VHF) Risk Clinical Lab Requisition Form 9

Example Laboratory Activation Checklist for Viral Hemorrhagic Fever (VHF) 10

Example Viral Hemorrhagic Fever (VHF) Pre-Analytic Cooler Receipt and Accessioning Procedural Checklist 14

Example Laboratory Procedural Checklists for Viral Hemorrhagic Fevers (VHFs) 16

Example Class II Biosafety Cabinet (BSC) Placemat for Analysis of Viral Hemorrhagic Fever (VHF) Specimens 25

Example Laboratory Test Result Form 27

Example Laboratory Infection Control Task Table Guidelines for Viral Hemorrhagic Fevers (VHFs) 28

Example Laboratory Donning and Doffing Viral Hemorrhagic Fevers (VHF) Personal Protective Equipment (PPE) Checklist 29

Example Room Entry Log 32

Example DOT Shipper’s Manifest Form for Division 6.2 Materials (Category A/Category B) 33

Example Viral Hemorrhagic Fever (VHF) Category A Waste Handling Checklist 34

#

# **Introduction**

## ****Intent****

**This appendix provides example documents and templates to enhance the *Laboratory Testing Toolkit for a Suspect Viral Hemorrhagic Fever (VHF) Patient*. These documents are intended to support facility-level planning for initial laboratory testing of a suspect viral hemorrhagic fever (VHF) patient. Pathogens that fall into this category include those that cause Crimean-Congo Hemorrhagic Fever (CCHF), Ebola Virus Disease (EVD), Lassa Fever, and Marburg Virus Disease (MVD).**

**This document does not include considerations for specimen collection processes, testing occurring at the bedside, or testing for non-VHF high consequence infectious diseases (HCIDs).**

## ****How to use this document****

**Facilities should amend the following example documents and templates to reflect institutional capabilities, capacities, and plans. Facilities should ensure that all documents incorporated into facility-level planning align with state and local health department plans. Highlighted text should be changed to reflect facility-level expectations and procedures.**

## ****Applicability and Scope****

**Guidance from your state and local health departments supersedes the information in these documents. The information included is intended to serve as a template to facilitate planning and preparedness activities related to initial laboratory testing of a suspect VHF patient.**

## ****Planning Assumptions****

- **Facilities will initiate the Identify-Isolate-Inform algorithm for a suspect VHF patient.**
- **This toolkit offers guidance on the management and processing of specimens in the laboratory setting and relies on the prior development of safe specimen collection procedures.**
- **State and CDC VHF specimen testing requires coordination with the state health department prior to specimen collection.**
- **It is expected that when a suspect VHF patient is identified at a facility, healthcare personnel (HCP) will be able to don appropriate Personal Protective Equipment (PPE) to assess the patient and collect specimens.**
- **All personnel who handle human specimens must comply with the** [Occupational Health and Safety Administration (OSHA) Bloodborne Pathogens Standard (29 CFR § 1910.1030)](https://www.osha.gov/pls/oshaweb/owadisp.show_document?p_id=10051&p_table=STANDARDS)**.**
- **Facilities are expected to have a plan to manage all waste generated in the management of a suspect VHF patient, which is considered Category A waste, until they have been determined to no longer meet criteria. Specimens collected from a suspect VHF patient must be packaged and shipped as Category A infectious substances in accordance with the** [Department of Transportation (DOT) Hazardous Materials Regulations (HMR) Title 49 Code of Federal Regulations (CFR) 173.196](https://www.ecfr.gov/current/title-49/subtitle-B/chapter-I/subchapter-C)**.**

# **Example Viral Hemorrhagic Fever (VHF) Laboratory Testing Menu**

##### Last reviewed: [date]; last updated: [date]

## Essential Tests

| Test | Order Code | Tube Type and Volume^1^ | Location & Instrument |
| --- | --- | --- | --- |
| Basic Metabolic Panel | [order code] | **Adult:** 3mL lithium heparin non-gel blood collection tube  **Pedi:** 1mL lithium heparin non-gel blood collection tube | Class II BSC,  Portable Blood Analyzer |
| Malaria Screen | [order code] | **Adult:** 3mL or 5mL whole blood (K_2_ EDTA) collection tube  **Pedi:** 1mL whole blood (K_2_ EDTA) collection tube | Class II BSC,  BinaxNOW POC Malaria Test |
| State Lab &  CDC PCR Samples^2^ | [order code] | **Adult:** 2 x 5mL whole blood (K_2_ EDTA) collection tube  **Pedi:** 2 x 1mL whole blood (K_2_ EDTA) collection tube | State Lab |
| Notes: 1. Use all tube types supplied in VHF Specimen Collection Supply Kit; 2. Two whole blood (K_2_ EDTA) collection tubes needed for State Lab and CDC specimens | | | |

## Additional Tests^2^

| Test | Order Code | Tube Type and Volume^1^ | Location & Instrument |
| --- | --- | --- | --- |
| Blood Culture | [order code] | **Adult:** Plastic adult blood culture set  **Pedi:** Plastic pediatric blood culture bottle | [location & instrument] |
| Blood Gas & Lactate^3^ | [order code] | **Adult:** 3mL lithium heparin non-gel blood collection tube  **Pedi:** 1mL lithium heparin non-gel blood collection tube | [location & instrument] |
| Liver Panel^3^ | [order code] | **Adult:** 3mL lithium heparin non-gel blood collection tube  **Pedi:** 1mL lithium heparin non-gel blood collection tube | [location & instrument] |
| CBC with 3-Part Differential | [order code] | **Adult:** 3mL whole blood (K_2_ EDTA) collection tube  **Pedi:** 1mL whole blood (K_2_ EDTA) collection tube | [location & instrument] |
| Extended Respiratory Viral Panel | [order code] | Viral transport medium (VTM) or universal transport medium (UTM) 3 mL tube with flocked NP swab | [location & instrument] |
| PT/INR | [order code] | Fresh non-anticoagulated whole blood (line) | [location & instrument] |
| Notes: 1. Use all tube types supplied in VHF Specimen Collection Supply Kit; 2. Recommended guidance for the performance of tests beyond the minimum recommended test menu are not included in this document - additional considerations with risk assessments are required prior to implementation. 3. Basic Metabolic Panel and Liver Panel may share 1 lithium heparin non-gel blood collection tube - additional lithium heparin non-gel blood collection tube needed for Blood Gasses & Lactate | | | |

# Example Viral Hemorrhagic Fever (VHF) Laboratory “Go Kit” Cart Packing List

A Laboratory “Go Kit” Cart, including par levels of appropriate equipment and supplies necessary to support activation of a VHF response. Cart contents should be tracked by laboratory personnel to ensure adequate supply and prevent use of expired products. Tracking also allows for contents to be rotated with the general laboratory consumables to minimize expiration before use.

## Lab “Go Kit” Cart Contents

| 🗹 | Count | Item | Order No. |
| --- | --- | --- | --- |
| 🞎 | 1 | 3-tier metal cart | [order code] |
| 🞎 | 4 | Disposable fluid-resistant or fluid-impervious gowns^1^ | [order code] |
| 🞎 | 1 ea. | Box of nitrile examination gloves (in all common sizes) | [order code] |
| 🞎 | 4 | Disposable eye protection^2^ | [order code] |
| 🞎 | 1 | Box of surgical masks^3^ | [order code] |
| 🞎 | 2 | Adult VHF Specimen Collection Supply Kit | [order code] |
| 🞎 | 2 | Pediatric VHF Specimen Collection Supply Kit | [order code] |
| 🞎 | 2 | Durable, leakproof secondary container for inter- and intra-facility specimen transport^4^ | [order code] |
| 🞎 | 2 | State Public Health Laboratory Specimen Submission Form (if applicable) | N/A |
| 🞎 | 6 | Biohazard container for benchtop waste (2 to be used specifically for Category A bench-top waste) | [order code] |
| 🞎 | 6 | Biohazard waste bags to double-line small biohazard container for benchtop waste (2 to be used specifically for Category A bench-top waste) | [order code] |
| 🞎 | 2 | Transport containers | [order code] |
| 🞎 | 1 | Small biohazard bench top waste container for sharps | [order code] |
| Notes: 1. Gowns should be solid-front, wrap-around, extend to mid-calf, and fluid-resistant (ANSI/AAMI BP70, Level 3) or fluid-impermeable (ANSI/AAMI BP70, Level 4); 2. Eye protection may be full-face shield, or goggles/safety glasses with side shield; 3. Surgical masks should be ASTM Level 3; 4. Recommend 2 coolers in different colors, one for samples to be sent to outside facilities, one for internal laboratory testing - these coolers should have pouches with the appropriate facility lab requisitions or state lab specimen submission forms | | | |

##

## Assembled Lab “Go Kit” Cart


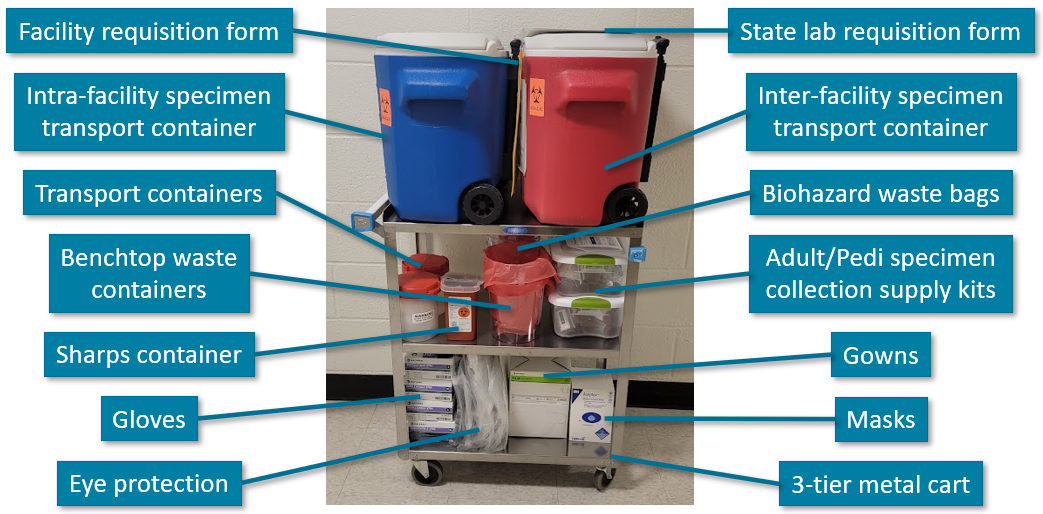


## Lab “Go Kit” Cart Contents


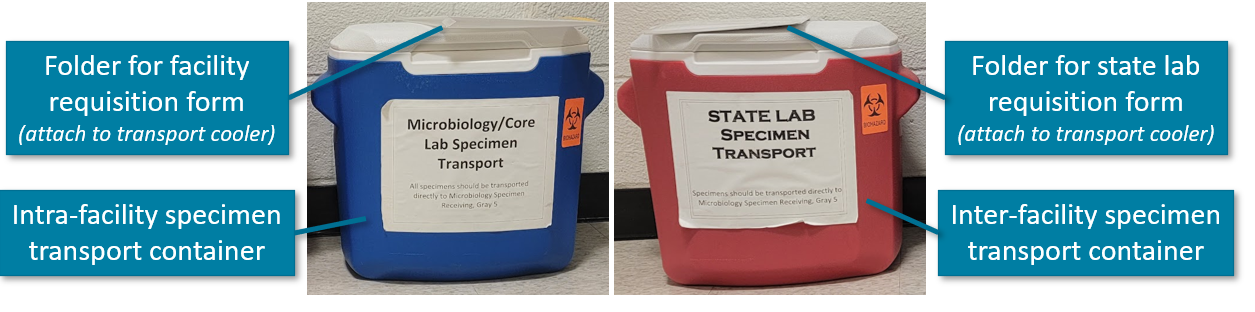


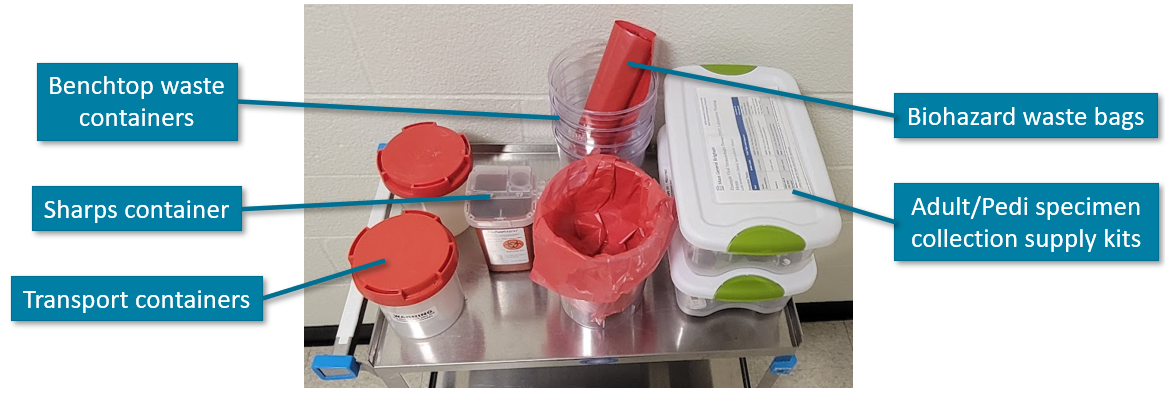


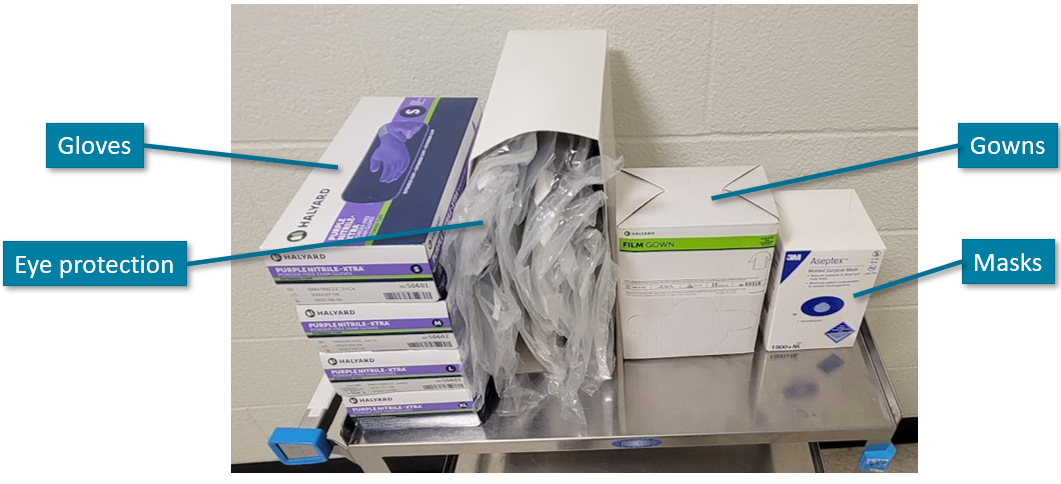


# Example Viral Hemorrhagic Fever (VHF) Specimen Collection Supply Kit Packing List

This document includes specimen collection kit contents based on the recommended minimum initial laboratory evaluations for a suspect VHF patient. These lists assume use of the BinaxNOW Point-of-Care (POC) Malaria Test. Supply lists should be adjusted based on instrument availability and selection.

## Specimen Collection Supply Kit Contents

| 🗹 | Count | Item | Order No. |
| --- | --- | --- | --- |
| 🞎 | 1 | VHF Laboratory Testing Menu | [order code] |
| 🞎 | 1 | Facility Laboratory Requisition Form | [order code] |
| 🞎 | 1 | State Public Health Laboratory Requisition Form | [order code] |
| 🞎 | 1 | Basic Metabolic Panel Specimen  **Adult:** 3mL lithium heparin non-gel blood collection tube  **Pedi:** 1mL lithium heparin non-gel blood collection tube | [order code] |
| 🞎 | 1 | Malaria Test Specimen (e.g., BinaxNOW POC Malaria Test)  **Adult:** 3mL or 5 mL whole blood (K_2_ EDTA) collection tube  **Pedi:** 1mL whole blood (K_2_ EDTA) collection tube | [order code] |
| 🞎 | 2 | State & CDC PCR Samples^1^  **Adult:** 5mL whole blood (K_2_ EDTA) collection tube  **Pedi:** 1mL whole blood (K_2_ EDTA) collection tube | [order code] |
| Notes: 1. Two whole blood (K_2_ EDTA) tubes needed for State Lab and CDC Specimens | | | |

## Other Supplies for Consideration^1^

| 🗹 | Count | Item | Order No. |
| --- | --- | --- | --- |
| 🞎 | 1 | Plastic Blood Culture Set  **Adult:** Plastic adult blood culture set (aerobic and anerobic)  **Pedi:** Plastic pediatric blood culture bottle (aerobic) | [order code] |
| 🞎 | 1 | Blood Gas & Lactate Test Specimen^2^  **Adult:** 3mL lithium heparin non-gel blood collection tube  **Pedi:** 1mL lithium heparin non-gel blood collection tube | [order code] |
| 🞎 | 0 | Liver Panel Test Specimen^2^  No additional supplies required – pull specimen from lithium heparin non-gel blood collection tube | [order code] |
| 🞎 | 1 | CBC with 3-part Differential  **Adult:** 3mL whole blood (K_2_ EDTA) collection tube  **Pedi:** 1mL whole blood (K_2_ EDTA) collection tube | [order code] |
| 🞎 | TBD | Research Study Samples | [order code] |
| 🞎 | 1 | Extended Respiratory Viral Panel  Viral transport medium (VTM) or universal transport medium (UTM) 3 mL tube with flocked NP swab | [order code] |
| 🞎 | 1 | PT/INR  Fresh non-anticoagulated whole blood (line) | [order code] |
| Notes: 1. Recommended guidance for the performance of tests beyond the minimum recommended test menu are not included in this document - additional considerations with risk assessments are required prior to implementation; 2. Basic Metabolic Panel and Liver Panel may share 1 lithium heparin non-gel blood collection tube - additional lithium heparin non-gel blood collection tube needed for Blood Gasses & Lactate | | | |

# Example Viral Hemorrhagic Fever (VHF) Risk Clinical Lab Requisition Form

| **Date Collected** |  | Place Patient Identification Label here:  *(Full name, MRN, CSN, Date of Birth required)* |
| --- | --- | --- |
| **Time Collected** |  |  |
| **Collected by** |  |  |
| **Specimen Source** | *(circle)* **Arterial**  or **Venus** |  |
| **Location** |  |  |
| **Ordering Provider** |  |  |

| 🗹 | **Test** | **Adult** | **Pediatric** |
| --- | --- | --- | --- |
| 🞎 | **Basic Metabolic Panel:**  Sodium, Potassium, Chloride, BUN, Creatinine, Anion Gap^1^, TCO2, Ionized Calcium, Glucose | 3mL lithium heparin non-gel blood collection tube | 1mL lithium heparin non-gel blood collection tube |
| 🞎 | **Malaria Screen** | 3mL or 5mL whole blood (K_2_ EDTA) collection tube | 1mL whole blood (K_2_ EDTA) collection tube |
| **Notes:** **1.** Calculated parameter  **Important:** The definitive identification of Ebola Zaire Virus requires additional testing and confirmation procedures in consultation with public health authorities. | | | |
| **Optional Tests^2^** | | | |
| 🞎 | **Blood Culture** | Plastic adult blood culture set | Plastic pediatric blood culture set |
| 🞎 | **Lactate & Blood Gases:**  (pH, PCO2, PO2, TCO2^1^, HCO3^1^,  SO2%^1^, BE^1^) | 3mL lithium heparin non-gel blood collection tube^4^ | 1mL lithium heparin non-gel blood collection tube^4^ |
| 🞎 | **Liver Function/Chemistry:**  Glucose, BUN, Creatinine, Calcium,  Albumin, Total Protein, ALT, AST,  Alkaline Phosphatase, Total Bilirubin | 3mL lithium heparin non-gel blood collection tube^3^ | 1mL lithium heparin non-gel blood collection tube^3^ |
| 🞎 | **CBC with 3-Part Differential** | 3mL whole blood (K_2_ EDTA) collection tube | 1mL whole blood (K_2_ EDTA) collection tube |
| 🞎 | **Extended Viral Respiratory Panel** | Viral transport medium (VTM) or universal transport medium (UTM) 3mL tube with flocked NP swab | |
| **Notes:** **1.** Calculated parameter; **2.** Recommended guidance for the performance of tests beyond the minimum recommended test menu are not included in this document **- a**dditional considerations with risk assessments are required prior to implementation **3.** Draw 1 lithium heparin non-gel blood collection tube for Basic Metabolic Panel and Liver Function Panel; **4.** Additional lithium heparin non-gel blood collection tube needed if Lactate & Blood Gases are ordered | | | |
| **CALL BEFORE DELIVERY (PHONE #)**  **HAND DELIVER IN [PREDETERMINED CONTAINER] TO [LOCATION] LABORATORY** | | | |
| LAB USE ONLY – PLACE SQ ACCESSION LABELS HERE  NOTE: TESTS BC, MGRP21, RMALA, AND BTCBCD MUST BE ACCESSIONED BY THE LAB. | | | |

# Example Laboratory Activation Checklist for Viral Hemorrhagic Fever (VHF)

The Laboratory Manager or Supervisor should assign appropriate personnel to the checklists included in the following table. Persons completing an assigned task list should report back to the Laboratory Manager with appropriate updates. Tasks should be initialed as they are completed. These checklists assume use of the BinaxNOW Point-of-Care (POC) Malaria Test and should be adjusted based on instrument availability and selection.

| **Activation Task List** | **Personnel** | **Assigned To** |
| --- | --- | --- |
| Notifications | Supervisor | [Name, Title] |
| Staffing | Supervisor | [Name, Title] |
| Lab Set Up | Technologist | [Name, Title] |
| Instrument QC | Technologist | [Name, Title] |

## Notifications Checklist

| **Task** | **Initial Below** |
| --- | --- |
| Contact the applicable state laboratory  State Public Health Lab (XXX-XXX-XXXX) |  |
| Notify internal laboratory leadership and stakeholders of VHF activation |  |
| Set up initial call with internal laboratory personnel to discuss situation and timeline to initial testing |  |
| Ensure initial response team is available |  |
| Assign technologist to Lab Set Up checklist (below) |  |
| Assign technologist to Instrument QC checklist (below) |  |
| [Insert additional steps as needed] |  |

## Staffing Checklist

| **Task** | **Initial Below** |
| --- | --- |
| Prepare staffing schedule for the upcoming 48-72 hours |  |
| Assign, at minimum, one laboratory technician for each shift |  |
| Assign a “Shipping Tech” for each shift |  |
| Ensure “Shipping Tech” is trained and certified in compliance with DOT or IATA requirements |  |
| Ensure all assigned personnel have demonstrated competency with donning and doffing appropriate PPE |  |
| Send initial staffing calendar to all staff |  |
| [Insert additional steps as needed] |  |

## Lab Set Up Checklist

Ensure the laboratory is stocked with enough supplies for 48-72 hours.

### Step 1: Prepare the laboratory space

| **Task** | **Initial Below** |
| --- | --- |
| Ensure BSC II is certified and functioning |  |
| Ensure electrical checks are up to date for all equipment |  |
| Empty all trash and biohazardous waste containers near the BSC II |  |
| Clear lab of any unnecessary items (e.g., boxes, lab coats, papers, etc.) |  |
| Ensure the lab “go kit” cart is stocked and located in the vicinity of the BSC II |  |
| [Insert additional steps as needed] |  |

### Step 2: Gather PPE

| **Count^1^** | **Item** | **Location** | **Initial Below** |
| --- | --- | --- | --- |
| 12 | Disposable fluid-resistant or -impervious gowns^2^ | Lab “go kit” cart |  |
| 1 ea. | Box of nitrile examination gloves (in most common sizes) | Lab “go kit” cart |  |
| 1 | Box of surgical masks^3^ | Lab “go kit” cart |  |
| 12 | Disposable eye protection^4^ | Lab “go kit” cart |  |
| **Notes: 1.** Materials count estimated for two lab techs per 12-hour shift; **2.** Gowns should be solid-front, wrap-around, extend to mid-calf, and fluid-resistant (ANSI/AAMI BP70, Level 3) or fluid-impermeable (ANSI/AAMI BP70, Level 4); **3.** Surgical masks should be ASTM Level 3; **4.** Eye protection may be full-face shield, or goggles/safety glasses with side shield | | | |

### Step 3: Gather general lab supplies

| **Count** | **Item** | **Location** | **Initial Below** |
| --- | --- | --- | --- |
| 2 | Absorbent pads (i.e., chux) | [Location] |  |
| 2 | Disposable bench-top waste container double-lined with biohazard waste bags – fill 1/3 full of disinfectant solution (e.g., Dispatch, Clorox Healthcare Bleach Germicidal) | [Location] |  |
| 1 | Small biohazard bench top sharps waste container | [Location] |  |
| 8 | Twist ties | [Location] |  |
| 1 | Container of facility-approved disinfectant wipes (e.g., Dispatch Wipes, Clorox Healthcare Bleach Germicidal Wipes) | [Location] |  |
| 6 | Isopropanol wipes | [Location] |  |
| 8 | Absorbent biohazard wipes (e.g., Fisherbrand BloodBloc Biohazard Wipes) | [Location] |  |
| 8 | Reinforced wiping cloths (e.g., WypAll) | [Location] |  |
| 1 | 70% Ethanol wash bottle | [Location] |  |
| 1 | Distilled water (DI) in squeeze bottle | [Location] |  |
| 10 | Extra specimen bags | [Location] |  |
| 1 | Biohazard-labeled specimen bag with absorbent materials | [Location] |  |
| 1 | Transport container for CDC and State Lab submissions | [Location] |  |
| *see note* | Extra tube caps or alternative (Note: 2 needed for **each** lithium heparin non-gel blood collection tube) | [Location] |  |
| 2 | Test tube racks | [Location] |  |
| 1 | Pencil | [Location] |  |
| 1 | Box of filtered pipette tips | [Location] |  |
| 1 | Adjustable pipette | [Location] |  |
| 2 | Microcentrifuge tubes | [Location] |  |
| 1 | Microcentrifuge tube stand | [Location] |  |
| 2 | Disposable transfer pipettes | [Location] |  |
| 1 | Roll of white labeling tape | [Location] |  |
| 1 | Cardboard Category A/B shipping box | [Location] |  |
| # | [Insert additional items as needed] | [Location] |  |

### Step 4: Gather portable blood analyzer system supplies

| **Count** | **Item** | **Location** | **Initial Below** |
| --- | --- | --- | --- |
| 1 | [Portable blood analyzer device] | [Location] |  |
| # | [Insert additional items as needed] | [Location] |  |
| # | [Item] | [Location] |  |
| # | [Item] | [Location] |  |
| # | [Item] | [Location] |  |

### Step 5: Gather Malaria point-of-care testing supplies

| **Count** | **Item** | **Location** | **Initial Below** |
| --- | --- | --- | --- |
| # | [List items required for Malaria point-of-care test (e.g., BinaxNOW POC Malaria Test)] | [Location] |  |
| # | [Item] | [Location] |  |
| # | [Item] | [Location] |  |
| # | [Item] | [Location] |  |

## Portable Blood Analyzer System QC Checklist

| **Step** | **Initial Below** |
| --- | --- |
| Check battery |  |
| Check temperature |  |
| [Insert additional steps as needed] |  |
| - [Step] |  |
| - [Step] |  |

# Example Viral Hemorrhagic Fever (VHF) Pre-Analytic Cooler Receipt and Accessioning Procedural Checklist

This checklist is intended to guide laboratory personnel in preparing transport supplies prior to specimen collection, receiving specimen coolers, and specimen accessioning. Use of this checklist assumes identification of a suspect viral hemorrhagic fever (VHF) patient.

## Initial Placement of Specimen Transport Coolers

| 🗹 | Step | |
| --- | --- | --- |
| 🞎 | Retrieve **Facility Specimen Cooler** and **CDC/State Lab Specimen Cooler** from [location] | |
| 🞎 | Ensure that each cooler is properly labeled and has a pouch attached to the handle for the appropriate requisition form (i.e., [*Viral Hemorrhagic Fever (VHF) Risk Clinical Lab Requisition Form*](#_Viral_Hemorrhagic_Fever) or appropriate state lab specimen submission form) | |
|  | **Facility Specimen Cooler**  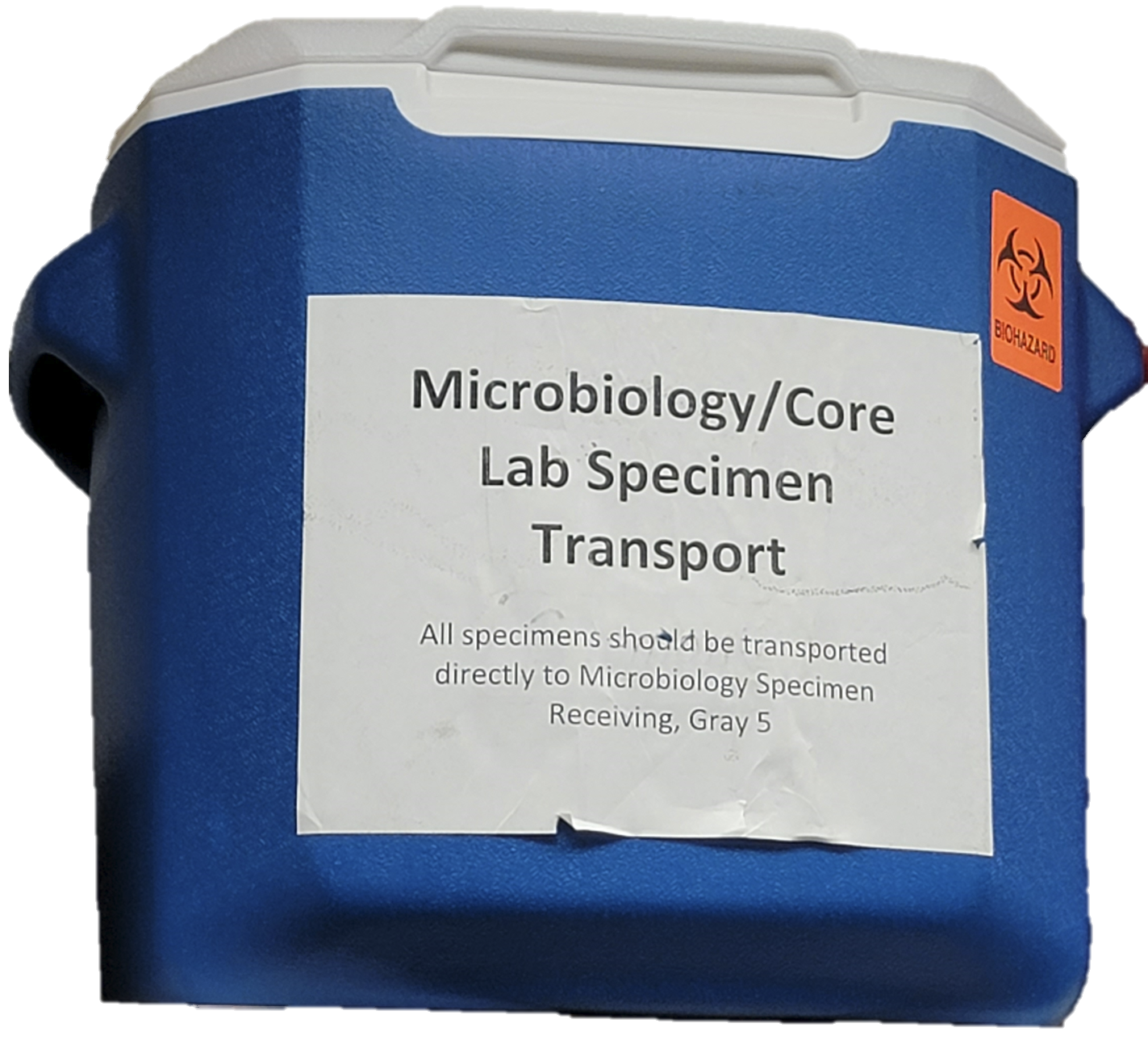 | **CDC/State Lab Specimen Cooler**  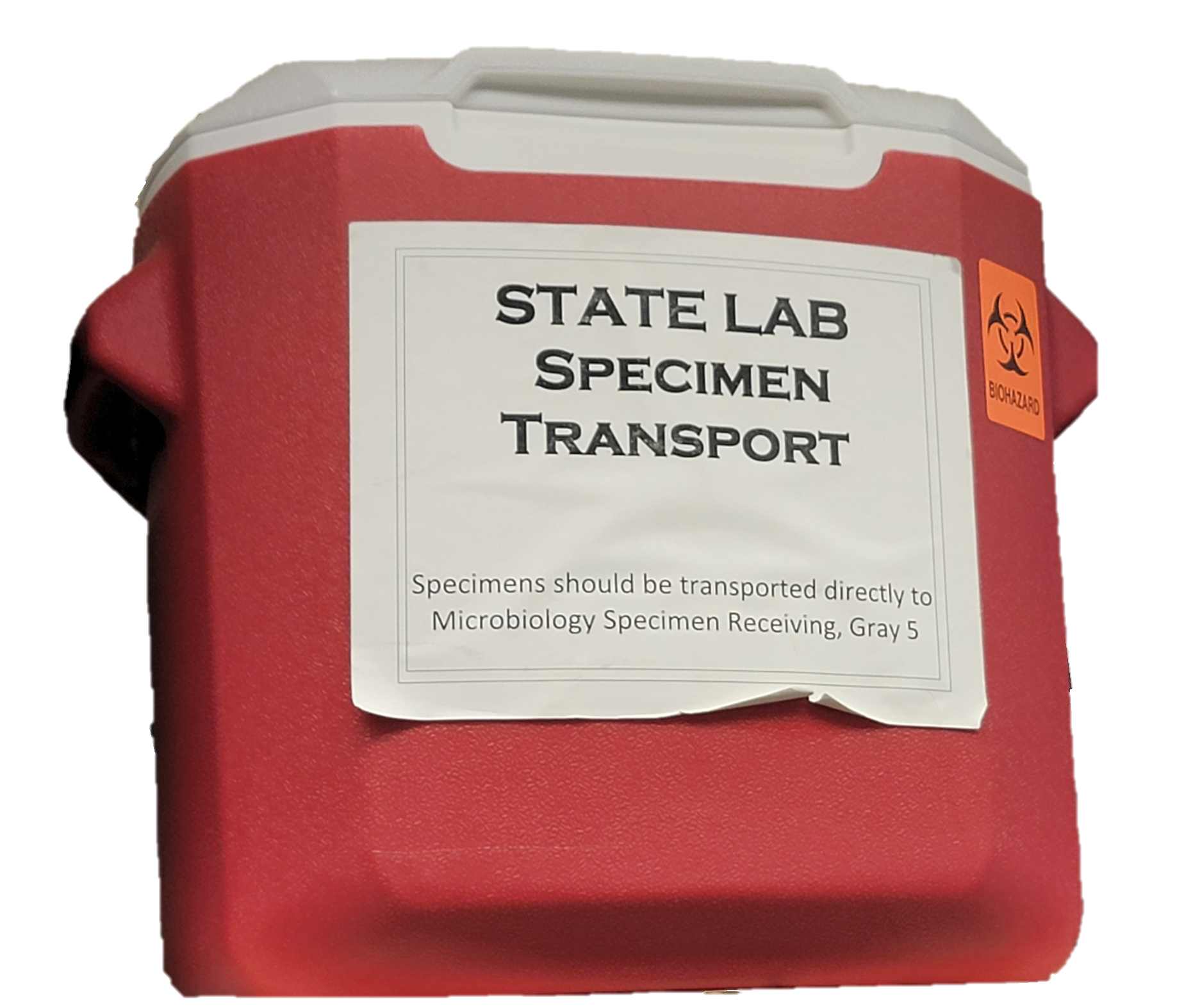 |
| 🞎 | [Pre-identified patient care team leadership role] will coordinate retrieval of the specimen transport coolers with [pre-identified laboratory leadership role] | |
| 🞎 | Add Specimen Collection Supply Kit (Adult or Pediatric) to **Facility Specimen Cooler** (see [*Viral Hemorrhagic Fever (VHF) Specimen Collection Supply Kit Packing List*](#_Example_Viral_Hemorrhagic_2) for details)  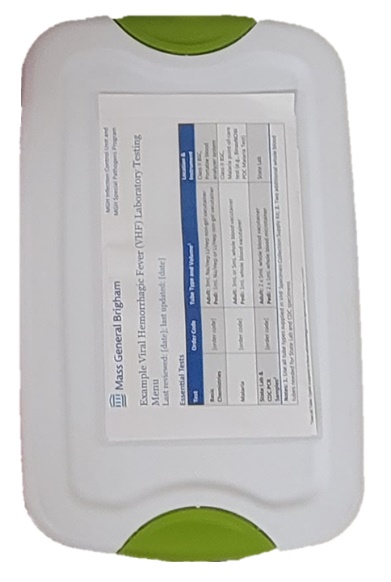 | |
| 🞎 | Add cold packs (found in [location] freezer) to the **CDC/State Lab Specimen Cooler** | |
| Note: Add portable coagulation testing device and applicable Job Aides to the Facility Specimen Cooler if used for bedside PT/INR | | |

## Receipt of Specimen Coolers

| 🗹 | Step |
| --- | --- |
| 🞎 | [Pre-identified laboratory leadership role] should notify laboratory staff at [phone/pager #] that testing has been approved |
| 🞎 | [Pre-identified patient care team leadership role] should notify the lab at [phone/pager #] when specimen coolers are on their way to the lab |
| 🞎 | Upon delivery of specimen coolers, provide staff with clean, empty **Facility Specimen Cooler** from [location] |
| 🞎 | Bring **Facility Specimen Cooler** and **CDC/State Lab Specimen Cooler** into the Specimen Receiving Area for accessioning |

## Accessioning of Specimens (Facility and CDC/State Lab Samples)

The Facility Specimen Cooler and CDC/State Lab Specimen Cooler should not be opened during the accessioning process.

| 🗹 | Step |
| --- | --- |
| 🞎 | Remove Lab Requisition Form from pouch on the **Facility Specimen Cooler** |
| 🞎 | Accession all marked tests as per laboratory protocols |
| 🞎 | Place labels on Lab Requisition Form and attach extra labels to form |
| 🞎 | Make two copies of the original Lab Requisition Form – place copies along with extra labels into **Facility Specimen Cooler** pouch |
| 🞎 | Remove State Lab Specimen Submission Form from pouch on **CDC/State Lab Specimen Cooler** |
| 🞎 | Accession specimens based on test requests on State Public Health Laboratory Specimen Submission Form |
| 🞎 | Place lab accession label on State Lab Submission Form – discard any extra labels |
| 🞎 | Make one copy of the original State Lab Submission Form – place original State Lab Submission Form into **CDC/State Lab Specimen Cooler** |
| 🞎 | Transport both coolers to [pre-identified lab] |

# Example Laboratory Procedural Checklists for Viral Hemorrhagic Fevers (VHFs)

The following checklists are intended to guide laboratory personnel through processes immediately before, during, and after analysis of confirmed or potential viral hemorrhagic fever (VHF) specimens. These lists assume use of the BinaxNOW Point-of-Care (POC) Malaria Test. Supply lists should be adjusted based on instrument availability and selection.

Use of this checklist assumes identification of a suspect VHF patient.

## Class II BSC Set-up for Specimen Preparation Checklist

Class II BSC preparation is performed by a **Lab Tech**.

If there is visible contamination on a surface at any point, **stop to clean and decontaminate the area or surface with a facility-approved disinfectant wipe** before moving on to the next step.

### Step 1: Gather the following materials needed OUTSIDE the BSC

| 🗹 | **Count** | **Material** | **Notes** |
| --- | --- | --- | --- |
| **🞎** | 1 | Timer |  |
| **🞎** | 2 | Prepped medical waste box |  |

### Step 2: Gather the following materials needed INSIDE the BSC

| 🗹 | **Count** | **Material** | **Notes** |
| --- | --- | --- | --- |
| **🞎** | 2 | Absorbent pads (i.e., chux) | Wet down absorbent side of pad (don’t soak) with facility-approved germicidal disinfectant prior to placing absorbent-side-up in BSC |
| **🞎** | 2 | Disposable bench-top waste container double-lined with biohazard waste bags and filled 1/3 full of disinfectant solution (e.g., Dispatch, Clorox Healthcare Bleach Germicidal) | Referred to as “VHF Waste” bench-top container |
| **🞎** | 1 | Small biohazard bench top sharps waste container |  |
| **🞎** | 8 | Twist ties | For sealing bench-top waste container |
| **🞎** | 1 | Container of facility-approved disinfectant wipes | Example: Dispatch Wipes or Clorox Healthcare Bleach Germicidal Wipes container (disinfectant must be found on EPA List L and List Q) |
| **🞎** | 6 | Isopropanol wipes |  |
| **🞎** | 8 | Absorbent biohazard wipes | Example: Fisherbrand BloodBloc Biohazard Wipes |
| **🞎** | 8 | Reinforced wiping cloths | Example: WypAll |
| **🞎** | 1 | 70% Ethanol wash bottle |  |
| **🞎** | 1 | Distilled water (DI) in squeeze bottle |  |
| **🞎** | 10 | Extra specimen bags |  |
| **🞎** | 1 | Biohazard-labeled specimen bag with absorbent materials |  |
| **🞎** | 1 | Transport container for CDC and State Lab submissions |  |
| **🞎** | *see note* | Extra tube caps or alternative | Note: 2 needed for **each** lithium heparin non-gel blood collection tube |
| **🞎** | 2 | Test tube racks |  |
| **🞎** | 1 | Pencil | For use as a stylus |
| **🞎** | 1 | Box of 200µL filtered pipette tips |  |
| **🞎** | 1 | 200µL adjustable pipette | Set at 95µL |
| **🞎** | 2 | Microcentrifuge tubes |  |
| **🞎** | 1 | Microcentrifuge tube stand |  |
| **🞎** | 2 | Disposable transfer pipettes |  |
| **🞎** | 1 | Box of 20µL (set to 15ml) |  |
| **🞎** | 1 | 20µL adjustable pipette |  |
| **🞎** | 1 | Roll of white labeling tape | To demarcate “clean” and “dirty” work areas |
| **🞎** | 1 | Rubber top for breaking control vials |  |
| **🞎** | 1 | Cardboard Category A/B shipping box |  |
| **🞎** | 1 | Portable blood analyzer machine |  |
| **🞎** | # | [items for portable blood analyzer] |  |
| **🞎** | # | [items for Malaria point-of-care test] |  |

### Step 3: Set up BSC workspace

| **🗹** | **Task** | **Notes** |
| --- | --- | --- |
| **🞎** | Perform and document BSC function checks | Check Magnehelic gauge reading and check airflow |
| **🞎** | Arrange materials inside BSC | See [*Class II Biosafety Cabinet (BSC) Placemat for Analysis of Viral Hemorrhagic Fever (VHF) Specimens*](#_Example_Class_II) for further details |
| **🞎** | Divide workspace into “clean” (left) and “dirty” (right) sides using white labeling tape to mark “CLEAN” and “DIRTY” sides |  |
| **🞎** | Initiate portable blood analyzer system updates according to the manufacturer’s instructions for use |  |
| **🞎** | Check supplies and material expiration dates |  |
| **🞎** | Perform daily maintenance and QC, if needed |  |

## CDC/State Lab Specimen Preparation Checklist

Preparation of CDC/State Lab specimens into Class II BSC is performed by a **Lab Tech** unless otherwise noted.

If there is visible contamination on a surface at any point, **stop to clean and decontaminate the area or surface with a facility-approved disinfectant wipe** before moving on to the next step.

| **🗹** | **Step** | **Notes** |
| --- | --- | --- |
| **🞎** | **Lab Tech:** Don PPE for work in Class II BSC | See [*Laboratory Donning and Doffing Personal Protective Equipment (PPE) Checklist for Viral Hemorrhagic Fevers (VHFs)*](#_Example_Laboratory_Donning) document for further instructions |
| **🞎** | Remove transport container from the **CDC/State Lab Specimen Cooler** and inspect for gross leakage | Container is translucent |
| **🞎** | **If specimens have leaked:** Discard directly into medical waste box and inform Lab Director On-Call (contact #) |  |
| **🞎** | Place transport container in “dirty” side of Class II BSC |  |
| **🞎** | Remove top from transport container |  |
| **🞎** | Remove specimen bag |  |
| **🞎** | Inspect bag for signs of specimen leakage |  |
| **🞎** | **If specimens are leaking, STOP - DO NOT TEST**  Discard directly into “VHF Waste” bench-top container AND contact Lab Director On-Call (contact #) and inform them |  |
| **🞎** | Open specimen bag |  |
| **🞎** | Remove one blood collection tube from specimen bag using a facility-approved disinfectant wipe |  |
| **🞎** | Clean all tube surfaces, including septum, with second facility-approved disinfectant wipe |  |
| **🞎** | Place tube in test tube rack and allow to sit for three minutes | **Set timer for 3 minutes** |
| **🞎** | Use 70% isopropanol pads to wipe off septum of blood collection tube |  |
| **🞎** | Discard disinfectant wipes in “VHF Waste” bench-top container |  |
| **🞎** | Repeat all steps for second blood collection tube | **Ensure all tubes sit for 3 minutes after being wiped with a facility approved disinfectant wipe – set timer** |
| **🞎** | Wipe down gloves with facility-approved disinfectant wipe |  |
| **🞎** | Repack CDC/State Lab Specimens into clean transport container |  |
| **🞎** | Replace top on transport container and screw tightly |  |
| **🞎** | Move sealed transport container to front of BSC intake grill |  |
| **🞎** | **Shipping Tech:** Place cardboard Category A/B shipping box |  |
| **🞎** | **Lab Tech:** Remove sealed transport container from BSC and place into cardboard Category A/B shipping box |  |
| **🞎** | **Shipping Tech:** Complete packaging and shipping per the CDC [Guidance for Specimen Collection, Transport, and Submission](https://www.cdc.gov/vhf/ebola/laboratory-personnel/specimens.html) |  |
| **🞎** | **Lab Tech:** Continue to “Specimen Preparation for Internal Processing” Checklist |  |

## Specimen Preparation for Internal Processing Checklist

Preparation of CDC/State Lab specimens is performed by a **Lab Tech** unless otherwise noted.

If there is visible contamination on a surface at any point, **stop to clean and decontaminate the area or surface with a facility-approved disinfectant wipe** before moving on to the next step.

| 🗹 | **Step** | **Notes** |
| --- | --- | --- |
| **🞎** | Remove transport container from the **Intra-facility Specimen Cooler** and inspect for gross leakage |  |
| **🞎** | **If specimens have leaked:** Discard directly into medical waste box and contact Lab Director On-Call (contact #) and inform them |  |
| **🞎** | Place transport container in “dirty” side of Class II BSC |  |
| **🞎** | Remove top from transport container |  |
| **🞎** | Remove specimen bag |  |
| **🞎** | Inspect bag for signs of specimen leakage |  |
| **🞎** | **If specimens are leaking, STOP - DO NOT TEST**  Discard directly into “VHF Waste” bench-top container AND contact Lab Director On-Call (contact #) and inform them |  |
| **🞎** | Open specimen bag |  |
| **🞎** | Remove one blood collection tube from specimen bag using a facility-approved disinfectant wipe |  |
| **🞎** | Clean all tube surfaces, including septum, with second facility-approved disinfectant wipe |  |
| **🞎** | Place tube in test tube rack and allow to sit for three minutes | **Set timer for 3 minutes** |
| **🞎** | Use 70% isopropanol pads to wipe off septum of blood collection tube |  |
| **🞎** | Discard disinfectant wipes in “VHF Waste” bench-top container |  |
| **🞎** | Repeat all steps for remaining blood collection tubes | **Ensure all tubes sit for 3 minutes after being wiped with a facility-approved disinfectant wipe – set timer** |
| **🞎** | Wipe down gloves with facility-approved disinfectant wipe |  |
| **🞎** | Close used transport container and place on **dirty** side of the BSC workspace |  |
| **🞎** | Discard specimen bags in “VHF Waste” bench-top container |  |
| **🞎** | Wipe down gloves with facility-approved disinfectant wipe |  |

## Specimen Analysis

Specimen analysis is performed in the Class II BSC by a **Lab Tech** unless otherwise noted. Facilities may choose to have a second lab technician, a **Buddy Tech**, in the lab and support by recording analysis results. **Important:** Technicians must wear PPE appropriate to the tasks they will perform. See [*Example Laboratory Infection Control Task Table Guidelines for Viral Hemorrhagic Fevers (VHFs)*](#_Example_Laboratory_Infection_1) for guidance.

If there is visible contamination on a surface at any point, **stop to clean and decontaminate the area or surface with a facility-approved disinfectant wipe** before moving on to the next step. Repeat tests if error codes are obtained; **do not repeat abnormal results.**

### Step 1: Portable Blood Analyzer

| 🗹 | **Step** | **Notes** |
| --- | --- | --- |
| **🞎** | Verify each specimen collection tube against the requisition and label information; then place each tube in the rack | **If any leakage is observed, DO NOT TEST - place bag in “VHF Waste” container and inform Laboratory Director** |
| **🞎** | Position requisition with labels for each sample type to enable scanning of patient IDs during resting | Requisition may be clipped to the back wall of the BSC, if desired |
| **🞎** | Place portable blood analyzer on chux pad |  |
| **🞎** | Turn on analyzer and prepare device for analysis according to manufacturer instructions |  |
| **🞎** | Mix lithium heparin non-gel tube 10x by gentle inversion |  |
| **🞎** | Remove the cap using a biohazard wipe and discard cap and biohazard wipe in the “VHF Waste” container |  |
| **🞎** | Place tube in specimen rack to pipette the sample |  |
| **🞎** | Using the pipette and tip, slowly draw up required blood sample | Ensure there are no bubbles or air gaps |
| **🞎** | Dispense blood sample according to manufacturer’s instructions |  |
| **🞎** | Eject tip into the sharps container |  |
| **🞎** | Using another biohazard wipe, fold the sample closure over the sample well and press the rounded end (not the center of pad) of the closure until it snaps in place - discard the biohazard wipe into “VHF Waste” container |  |
| **🞎** | Position portable blood analyzer on chux pad so that next steps can be performed without moving the instrument |  |
| **🞎** | Perform analysis according to manufacturer instructions |  |
| **🞎** | The Lab Tech will read the results from the portable blood analyzer to the Buddy Tech who will record them on the manual Laboratory Test Results Sheet and **read back** the results to the Lab Tech to verify | Use page arrow to view multiple results page |

### Step 2: BinaxNOW Point-of-Care (POC) Malaria Test

| 🗹 | **Step** | **Notes** |
| --- | --- | --- |
| **🞎** | Wipe down gloves with bleach wipe then wipe down gloves with a 70% Ethanol-soaked wipe |  |
| **🞎** | Perform rapid malaria testing according to procedure |  |
| **🞎** | Remove the test device from the foil pouch just prior to use |  |
| **🞎** | Open the device and lay it flat on the work surface |  |
| **🞎** | Using absorbent biohazard wipe (e.g., Fisherbrand BloodBloc Biohazard Wipe), mix whole blood (K_2_ EDTA) blood collection tube gently |  |
| **🞎** | Using absorbent biohazard wipe (e.g., Fisherbrand BloodBloc Biohazard Wipe), remove blood collection tube stopper |  |
| **🞎** | Discard stopper in “VHF Waste” container |  |
| **🞎** | Use a 200µL adjustable pipette with a filtered pipet tip prime the pipette tip by drawing up the blood sample and expelling it a few times, then slowly draw up 15µL of blood into the pipette |  |
| **🞎** | Slowly apply it to the bottom half of the PURPLE sample pad |  |
| **🞎** | Discard the used pipet tip into the red sharps container |  |
| **🞎** | Hold Reagent A bottle upside down vertically and add two (2) free-falling drops of Reagent A to white pad immediately below the purple sample pad, allowing the first drop to absorb into the pad before adding the second drop  **Do NOT add Reagent A directly to the purple pad** | **NOTE:** To ensure delivery of the appropriate volume of Reagent A to both pads of the test device, hold the vial vertically ½ - 1 inch above the pads and slowly add free flowing drops. |
| **🞎** | While waiting for blood to flow up the strip, place clean blood collection tube top on patient's blood collection tube using absorbent biohazard wipe (e.g., Fisherbrand BloodBloc Biohazard Wipe) |  |
| **🞎** | Allow the blood sample to run up the length of the test strip  **Do NOT allow the blood to run into or under the absorbent pad at the TOP of the strip** | **NOTE:** If blood flow appears to pause or is less than halfway up the strip after one minute, add **one** additional drop of Reagent A to the white pad at the bottom of the test strip |
| **🞎** | Just before the sample reaches the base of the white absorbent pad at the top of the test strip, **SLOWLY** add four (4) free-falling drops of Reagent A to the wash pad at the top left-hand side of the test device, allowing each drop to absorb into the pad before adding the next | **NOTE:** The third and fourth drops may not completely absorb into the pad |
| **🞎** | When the sample just reaches the base of the white absorbent pad at the top of the test strip, remove the adhesive liner from the right edge of the device, and close the device allowing the Reagent A to wash (clear) the blood sample off the test strip | **NOTE:** To ensure good devise closure and test flow, press firmly along the entire edge of the right of the result window |
| **🞎** | Set the timer for 15 minutes and start timer |  |
| **🞎** | Read the test result 15 minutes after closing the test device - results read before or after 15 minutes may be inaccurate | **NOTE**: When reading test results, tilt the device to reduce glare in the result window, if necessary |
| **🞎** | The Lab Tech will read the results to the Buddy Tech who will record them on the manual Laboratory Test Results Sheet |  |
| **🞎** | Lab Tech will discard BinaxNOW Malaria Rapid Test card into “VHF Waste” container |  |
| **🞎** | **Lab Tech:** Proceed to “Post-Analytical Checklist” |  |

## Post-Analytical Checklist

The “Post-Analytical Checklist” is performed by a **Lab Tech** unless otherwise noted.

If there is visible contamination on a surface at any point, **stop to clean and decontaminate the area or surface with a facility-approved disinfectant wipe** before moving on to the next step.

| 🗹 | **Step** | **Notes** |
| --- | --- | --- |
| **🞎** | Once results are verified, use biohazard wipes to discard specimen tubes into “VHF Waste” container |  |
| **🞎** | Place requisition and any extra labels into “VHF Waste” container |  |
| **🞎** | Remove top layer gloves and place into “VHF Waste” container |  |
| **🞎** | Prepare “VHF Waste” container for disposal by lifting inner waste bag from the clean side and pull up slowly until clean side of bag is fully exposed |  |
| **🞎** | Twist bag to seal and then secure with twist tie |  |
| **🞎** | Repeat above step with outer waste bag |  |
| **🞎** | Wipe down exterior surfaces of instruments (including portable blood analyzer docking station, pipette, and stylus/pencil) with disinfectant wipes | Make sure to carefully clean all areas where you touch the instrument |
| **🞎** | Wait 3 minutes | **Set timer** |
| **🞎** | Use deionized water-soaked wiping cloth to remove residue from all surfaces cleaned with disinfectant |  |
| **🞎** | Wipe down sharps container with disinfectant wipes and place container into biohazard waste bag |  |
| **🞎** | Place clean portable blood analyzer back on the clean docking station |  |
| **🞎** | Fold up chux pad and place in a biohazard waste bag |  |
| **🞎** | Wipe down work surfaces, back wall, and view screen of BSC with disinfectant wipes |  |
| **🞎** | Disinfect the empty transport container and lid, inside and out, with disinfectant wipes |  |
| **🞎** | Wait three minutes | **Set timer** |
| **🞎** | Use 70% ethanol-soaked wiping cloth to remove residue from all surfaces cleaned with disinfectant, including the transport container and lid |  |
| **🞎** | Allow to dry |  |
| **🞎** | Twist biohazard waste bag to seal and then secure with twist tie |  |
| **🞎** | Doff PPE using Doffing PPE Checklist as a guide - make sure PPE is discarded into a medical waste box |  |

#

# Example Class II Biosafety Cabinet (BSC) Placemat for Analysis of Viral Hemorrhagic Fever (VHF) Specimens


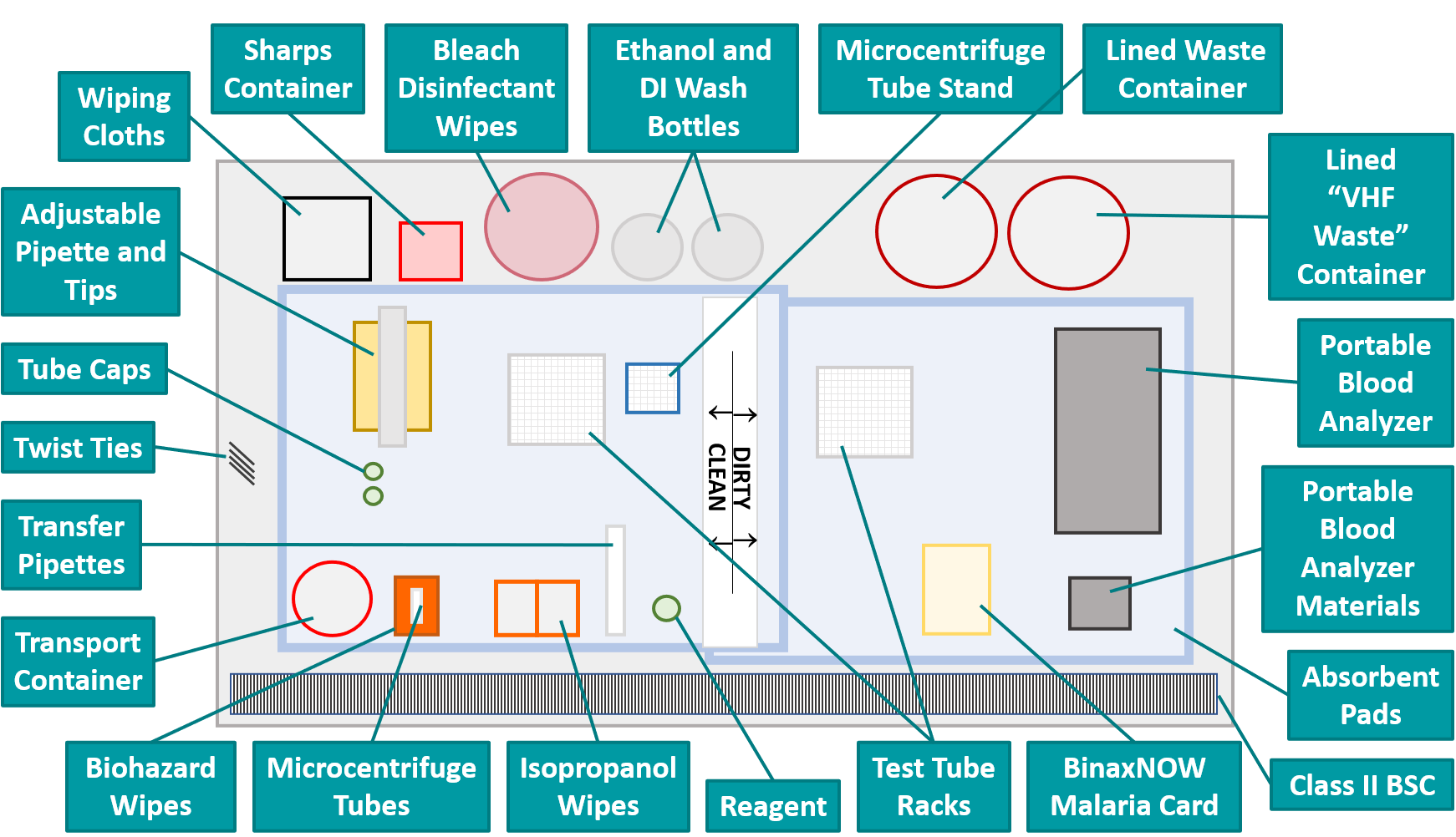


## Reference Image


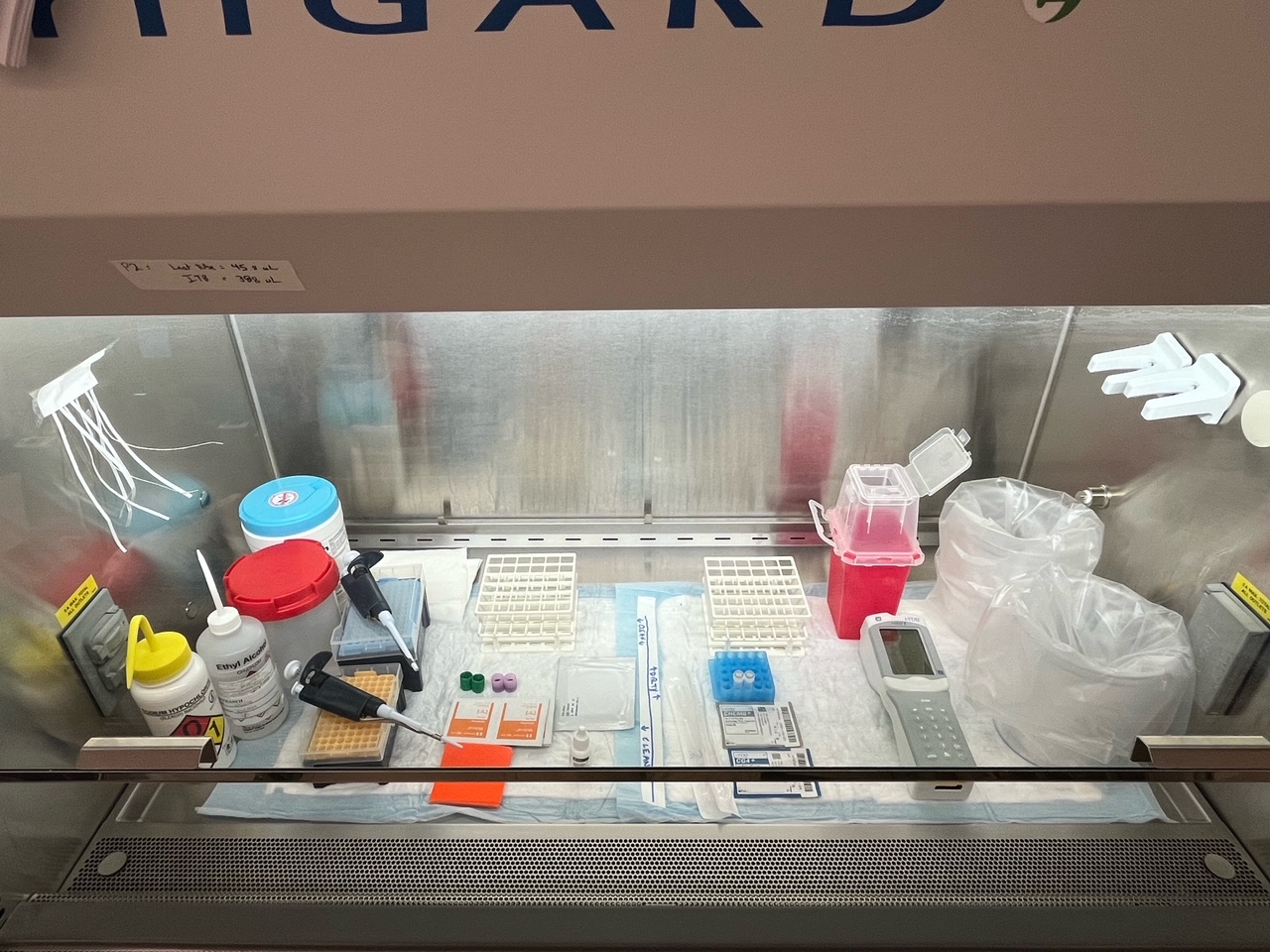


# Example Laboratory Test Result Form

This example results form should be modified to reflect internal capabilities.

| Patient Information: | Apply Patient **Requisition** Label: |
| --- | --- |
| NAME: | Circle sample type: **Arterial** or **Venous** |
| MRN: |  |
| CSN: |  |
| DATE/TIME: |  |

| **CALL priority analytes to [predetermined location]: [phone #]** |
| --- |

| **i-STAT CG4+** | | | | | |  | **i-STAT CHEM8+** | | | | | |
| --- | --- | --- | --- | --- | --- | --- | --- | --- | --- | --- | --- | --- |
| Patient ID: Scan **CSN** or manually enter "AC" then CSN | | | | | |  | Patient ID: Scan **CSN** or manually enter "AC" then CSN | | | | | |
|  | Results | Units | Ref Range | Called |  |  |  | Results | Units | Ref Range | Called |  |
| **pH** |  | X | 7.31-7.41 |  |  |  | **Na+** |  | mmol/L | 138-146 |  |  |
| **pCO2** |  | mmHg | 35-45 |  |  |  | **K+** |  | mmol/L | 3.5-4.9 |  |  |
| **pO2** |  | mmHg | 80-105 |  |  |  | **Cl-** |  | mmol/L | 98-109 |  |  |
| **BE*** |  | mmol/L | (-2)-(+3) |  |  |  | **iCa** |  | mmol/L | 1.12-1.32 |  |  |
| **HCO3*** |  | mmol/L | 22-26 |  |  |  | **TCO2** |  | mmol/L | 23-27 |  |  |
| **TCO2*** |  | mmol/L | 23-27 |  |  |  | **GLU** |  | mg/dL | 70-105 |  |  |
| **SO2%*** |  | % | 95-98 |  |  |  | **BUN** |  | mg/dL | 8-26 |  |  |
| **LAC** |  | mmol/L | 0.36-1.25 |  |  |  | **CREA** |  | mg/dL | 0.6-1.3 |  |  |
| *Value is calculated parameter | | | | | |  | **HCT** |  | %PVC | 38-51 |  |  |
|  | | | | | |  | **HBG*** |  | g/dL | 12-17 |  |  |
| **POCHi (BTCBCD)** | | | | | |  | **AN GAP*** |  | mmol/L | 7-22 |  |  |
| Patient ID: Scan **CID** or manually enter without letter | | | | | |  | *Value is calculated parameter | | | | | |
|  | Results | Units | Ref Range | Called |  |  |  | | | | | |
| **WBC** |  | K/uL | 4.5-11.0 |  |  |  | **PICCOLO (BTCM10)** | | | | | |
| **RBC** |  | M/uL | 4.50-5.90 |  |  |  | Patient ID: Manually enter the CSN | | | | | |
| **HGB** |  | g/dL | 13.5-17.5 |  |  |  |  | Results | Units | Ref Range | Called |  |
| **HCT** |  | % | 41.0-53.0 |  |  |  | GLU |  | mg/dL | 70-110 |  |  |
| MCV |  | fL | 80.0-100.0 |  |  |  | BUN |  | mg/dL | 8-25 |  |  |
| MCH |  | pg | 26.0-34.0 |  |  |  | CREAT |  | mg/dL | 0.6-1.5 |  |  |
| MCHC |  | g/dL | 31.0-37.0 |  |  |  | UA | Do not report/not validated | | | | |
| **PLT** |  | K/uL | 150-400 |  |  |  | CA |  | mg/dL | 8.5-10.5 |  |  |
| LYMPH% |  | % | 22-44 |  |  |  | ALB |  | g/dL | 3.3-5.0 |  |  |
| MXD% | Do not report/not in Epic | | | | |  | TP |  | g/dL | 6.0-8.3 |  |  |
| NEUT% |  | % | 40-70 |  |  |  | ALT |  | U/L | 10-55 |  |  |
| LYMPH# |  | K/uL | 1.0-4.8 |  |  |  | AST |  | U/L | 10-40 |  |  |
| MXD# | Do not report/not in Epic | | | | |  | ALP |  | U/L | F: 23-78 |  |  |
| NEUT# |  | K/uL | 1.8-7.7 |  |  |  |  |  |  | M: 35-90 |  |  |
| RDW-SD | Do not report/not in Epic | | | | |  | TBILI |  | mg/dL | 0.0-1.0 |  |  |
| RDW-CV |  | % | 11.5-14.5 |  |  |  | GGT | Do not report/not validated | | | | |
| MPV |  | fL | 8.4-12.0 |  |  |  | AMY | Do not report/not validated | | | | |

| Results called by: |  |  | Lab Tech: |  |
| --- | --- | --- | --- | --- |
| Results called to: |  |  | Buddy Tech: |  |
| Date/time called: |  |  | NOTES: | |

# Example Laboratory Infection Control Task Table Guidelines for Viral Hemorrhagic Fevers (VHFs)

## Personal Protective Equipment (PPE) Descriptions

The following chart includes descriptions of the personal protective equipment (PPE) included in the task table. **All PPE should be single use/disposable.**

| Standard Laboratory Personal Protective Equipment (PPE) | |
| --- | --- |
| Laboratory Coat | **Nitrile Exam Gloves** |
| 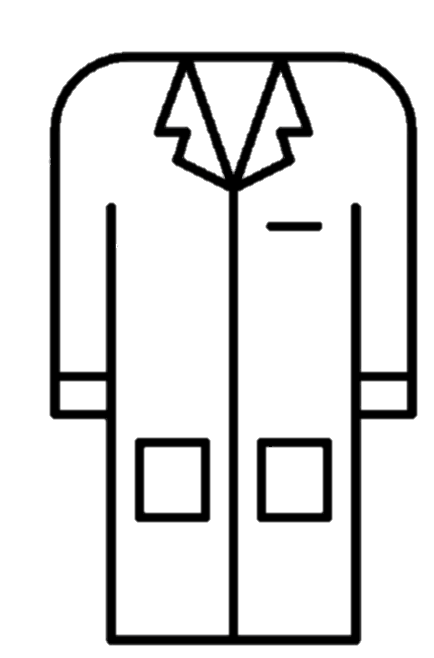 | 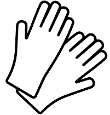 |

| Class II Biosafety Cabinet (BSC) Viral Hemorrhagic Fever (VHF) Personal Protective Equipment (PPE) | | | |
| --- | --- | --- | --- |
| Gown | **Gloves** | **Eye Protection** | **Surgical Mask** |
| 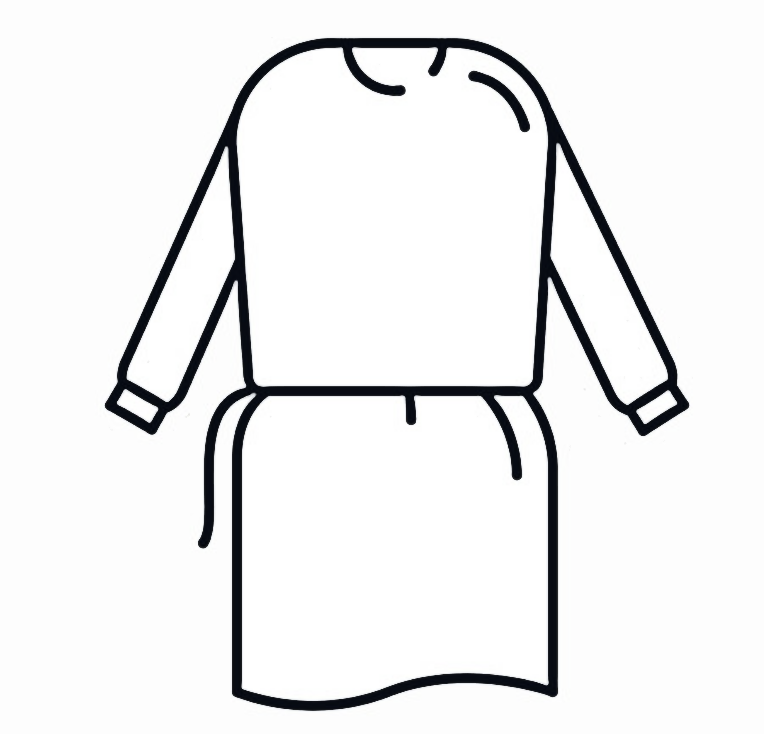 | 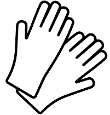 | 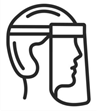 | 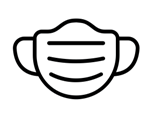 |
| Gowns should be solid-front, wrap-around, extend to mid-calf, and fluid-resistant (ANSI/AAMI BP70, Level 3) or fluid-impermeable (ANSI/AAMI BP70, Level 4) | Gloves should be nitrile examination gloves | Eye protection may be full-face shield, or goggles/safety glasses with side shield | Surgical masks should be ASTM Level 3 |

## Infection Control Task Table Guidelines

| Task | PPE | Location |
| --- | --- | --- |
| Transporting VHF-related specimens in a durable, leakproof secondary container that has been appropriately disinfected | No PPE should be worn |  |
| Handling VHF specimens for receiving and accessioning | Standard Lab PPE |  |
| Opening evacuated VHF-related specimen containers | Class II BSC VHF PPE | Class II BSC |
| Decanting or transferring of VHF-related specimens or contaminated fluids to other vessels for testing or storage | Class II BSC VHF PPE | Class II BSC |
| VHF-related specimen processing | Class II BSC VHF PPE | Class II BSC |
| Cleaning of VHF-related specimen spills and/or VHF-related specimen spills when sharps are present | Class II BSC VHF PPE |  |
| Disposal of VHF-related waste and contaminated equipment | Class II BSC VHF PPE |  |
| Packaging VHF-related specimens for shipment to an outside facility | Class II BSC VHF PPE |  |
| Clerical functions in lab area where VHF-related specimens are being processed and analyzed (no interaction with BSC) | Standard Lab PPE |  |

# Example Laboratory Donning and Doffing Viral Hemorrhagic Fevers (VHF) Personal Protective Equipment (PPE) Checklist

##### last reviewed: [date]; last updated: [date]

## Donning Personal Protective Equipment (PPE) Preparation

**All PPE should be single use/disposable.** Ensure the following PPE is available for each laboratory personnel donning PPE **in their required size**:

- 2 pair of nitrile examination gloves
- 1 gown that is solid-front, wrap-around, extend to mid-calf, and fluid-resistant (ANSI/AAMI BP70, Level 3) or fluid-impermeable (ANSI/AAMI BP70, Level 4)
- 1 surgical mask (ASTM Level 3)
- 1 full-face shield or goggles/safety glasses with side shield

Identify a **Trained Observer** to monitor and coach the donning procedures. The **Trained Observer** will read the following checklist verbatim and will ensure the donning process is done slowly, correctly, without distraction, and that all protocols are adhered to.

## Donning PPE Checklist

| 🗹 | **Step** |
| --- | --- |
| **🞎** | I am the **Trained Observer**. The donning process is conducted under my supervision. I will read aloud each step of the procedure and confirm the integrity of the ensemble when you are done. |
| **🞎** | Remove all personal items (e.g., jewelry, watches, pagers, etc.). |
| **🞎** | Visually inspect the PPE ensemble to be worn to ensure it is in serviceable condition, all required PPE and supplies are available, and the sizes are correct for the healthcare personnel. |
| **🞎** | Perform hand hygiene with alcohol-based hand rub (ABHR) - continue rubbing hands until dry. |
| **🞎** | Put on first pair of nitrile examination gloves. |
| **🞎** | Put on gown and ensure cuffs of inner gloves are tucked under the gown sleeves and hook gown loops over thumb or middle finger. |
| **🞎** | To secure the gown, first tie the inner gown tie, then the outer tie. **Trained Observer** may assist with this. Ensure gown is tied securely and allows for unrestricted movement. |
| **🞎** | Don surgical mask. |
| **🞎** | Put on second pair of nitrile examination gloves. Ensure cuffs are pulled over gown sleeves. |
| **🞎** | Put on full-face shield or goggles/safety glasses with side shield. |
| **🞎** | Now that you have completed the donning process, I (**Trained Observer**) will verify the integrity of the ensemble. You should be comfortable and able to complete a range of motions. |
| **🞎** | Perform hand hygiene with ABHR. Continue rubbing hands until gloves are dry. |

## Doffing PPE Preparation

To facilitate the doffing process, ensure a medical waste box is near the Class II BSC.

Identify a **Trained Observer** and **Doffing Buddy**. The **Trained Observer** will verbalize and monitor the doffing procedures according to the checklist. The **Trained Observer** should stand six feet away and will read the following checklist verbatim and will ensure the doffing process is done slowly, correctly, without distraction, and that all protocols are adhered to. The **Doffing Buddy** will assist as needed with the removal of personal protective equipment (PPE). The **Doffing Buddy** should don task-appropriate PPE as defined in the [*Laboratory Infection Control Task Table Guidelines for Viral Hemorrhagic Fevers (VHFs)*](#_Example_Laboratory_Infection_1).

## Doffing PPE Checklist

| 🗹 | **Step** |
| --- | --- |
| **🞎** | I am the **Trained Observer**. The doffing process is conducted under my supervision. I will read aloud each step of the procedure and confirm visually that the personal protective equipment (PPE) has been removed properly. Do not begin actions until I have finished reading the step. Remember to avoid reflexive actions that may put you at risk, such as touching your face. Your hands should remain out and away from your body. |
| **🞎** | **Prior to removing your hands from the BSC**, disinfect **outer-gloved** hands with either a facility-approved disinfectant wipe or alcohol-based hand rub (ABHR). Let gloves dry completely before moving to next step. |
| **🞎** | Remove hands from BSC and inspect outer-layer gloves for visible contamination, cuts, or tears. If visibly contaminated disinfect using a facility-approved disinfectant wipe |
| **🞎** | Remove **outer-layer** glove, taking care not to contaminate inner-layer glove:   - grasp outside of glove cuff at wrist with the opposite gloved hand - slowly peel glove off hand, turning it inside out - do not discard |
| **🞎** | Use inside-out glove to remove **outer-layer** glove on the other hand, taking care not to contaminate inner-layer glove:   - grasp outside of glove near cuff at wrist with the inside-out first glove - slowly peel glove off hand, turning it inside out |
| **🞎** | Discard both outer-layer gloves into medical waste container |
| **🞎** | Inspect **inner-layer** gloves to assess for visible contamination, cuts, or tears. If visibly contaminated, cut, or torn, disinfect using a facility-approved disinfectant wipe and notify the Lab Manager to review occupational exposure risk per protocol. |
| **🞎** | Disinfect **inner-layer** gloves with either a facility-approved disinfectant wipe or ABHR. Let gloves dry completely before moving to next step. |
| **🞎** | Remove full-face shield by tilting your head slightly forward, grabbing the rear strap, and pulling it over your head, gently allowing the full-face shield to fall forward and discard. Avoid touching the front surface of the full-face shield. |
| **🞎** | Disinfect **inner-layer** gloves with either a facility-approved disinfectant wipe or ABHR. Let gloves dry completely before moving to next step. |
| **🞎** | The **Doffing Buddy** will undo the first gown tie, then the second |
| **🞎** | Slowly grasp the gown at the shoulders and pull the gown forward and down over the arms and gloved hands |
| **🞎** | Holding your arms away from your body, fold the gown so that the outside of the gown is folded in. Be careful not to touch the outside of the gown. The **Doffing Buddy** may assist as needed. |
| **🞎** | Discard gown into medical waste box |
| **🞎** | Disinfect **inner-layer** gloves with either a facility-approved disinfectant wipe or Alcohol Based Hand Rub (ABHR). Let gloves dry completely before moving to next step. |
| **🞎** | Remove surgical mask by tilting your head slightly forward, grasping the elastic strap, and remove without touching the front of the mask. Discard surgical mask in medical waste box. |
| **🞎** | Disinfect **inner-layer** gloves with either a facility-approved disinfectant wipe or Alcohol Based Hand Rub (ABHR). Let gloves dry completely before moving to next step. |
| **🞎** | Remove **inner-layer** glove, taking care not to contaminate bare hands:   - grasp outside of glove cuff at wrist with the opposite gloved hand - slowly peel glove off hand, turning it inside out - do not discard |
| **🞎** | Use inside-out glove to remove **inner-layer** glove on the other hand, taking care not to contaminate bare hands:   - grasp outside of glove near cuff at wrist with the inside-out first glove - slowly peel glove off hand, turning it inside out |
| **🞎** | Discard both inner-layer gloves into medical waste box |
| **🞎** | Perform hand hygiene with ABHR. Continue rubbing hands until dry. |
| **🞎** | **Trained Observer:** Perform a final inspection of laboratory personnel for any indication of contamination. If contamination is identified, immediately inform the **Lab Manager**, and await further direction before exiting doffing area. |

# Example Room Entry Log

Room entry management is the responsibility of the Laboratory Manager or other local delegate:

- Start a new log every morning at 0700
- Affix ADT sticker only after log is taken down to provide patient confidentiality
- FAX this log every morning to the Occupational Health Department: [fax #]

| **Date:** | *Place Patient ADT sticker here before faxing*  *and after log no longer posted* |
| --- | --- |
| **Room Number/Location:** |  |
| **Page ____ of ____** |  |

| **Healthcare Personnel Name** | **Contact Email & Phone Number** | **Employee ID** |
| --- | --- | --- |
| *Example: Santa Claus* | *Example: sclaus@mgh.harvard.edu, 617-726-2000* | *Example: 0123456789* |
|  |  |  |
|  |  |  |
|  |  |  |
|  |  |  |
|  |  |  |
|  |  |  |
|  |  |  |
|  |  |  |
|  |  |  |
|  |  |  |
|  |  |  |
|  |  |  |
|  |  |  |
|  |  |  |
|  |  |  |
|  |  |  |

# Example DOT Shipper’s Manifest Form for Division 6.2 Materials (Category A/Category B)

| **SHIPPER’S NAME & ADDRESS**  *(submitting facility to complete)* | | |
| --- | --- | --- |
| **Consignee:** | | |
| **Street:** | | |
| **City:** | **State:** | **Zip Code:** |

**(Please circle the type of box you are shipping and list the number of boxes below)**

| **# Boxes** | **Basic Description**  *UN #, Proper Shipping Name, Hazard Class* | **Total Quantity**  *(i.e., gm or ml)* |
| --- | --- | --- |
| **1** | UN2814, Infectious substance, affecting humans (suspected category A infectious substance), 6.2 |  |
|  | **-or-** |  |
| **1** | UN3373, Biological substance, affecting humans (Category B), 6.2 |  |
|  |  |  |
| **24 hr. Emergency Contact Phone:** *(include area code)* **____________________**    **Offeror’s Name or Contact #:** *(complete only if shipper is NOT the emergency contact)*  **_______________________________________________________** | | |

This is to certify that the above-named materials are properly classified, described, packaged, marked, and labeled, and are in proper condition for transportation according to the applicable regulations of the Department of Transportation.

**SHIPPER’S NAME: _________________________**

**SIGNATURE: ____________________________**

**DATE: ________________**

# Example Viral Hemorrhagic Fever (VHF) Category A Waste Handling Checklist

##### last reviewed: [date] ; last updated: [date]

## Category A Waste Removal Overview

All Category A waste will be placed in a plastic biohazard waste tub lined with two biohazard waste bags. Bags will not be allowed to be overfilled; the inner biohazard waste bag will be removed and replaced when ½ full. The laboratory will contact the facility’s Environmental Services Department for removal; an Environmental Services Manager will oversee and manage all Category A waste removal from the laboratory.

Facilities may choose to sequester waste pending CDC confirmatory testing. Waste sequestration requires facilities to dedicate a secured, access-controlled room. If a VHF has been ruled-out, waste can be managed routinely according to the appropriate pathogen classification standards. If a VHF or other Category A pathogen is confirmed, waste disposal should follow your facility’s Category A waste disposal protocol. The waste removal processes below assumes that an autoclave is not present and a vendor has been contracted for Category A waste removal from the healthcare facility for inactivation.

## Waste Removal from Biosafety Cabinet (BSC) To Biohazard Waste Tub

| 🗹 | **Personnel** | **Step** | |
| --- | --- | --- | --- |
| **🞎** | Observer | Record the following information:  **Lab Tech:** ______________________  **Date:** __________ | **Observer:** ______________________  **Time:** __________ |
| **🞎** | Lab Tech, Observer | Ensure **Lab Tech** has donned PPE according to the [*Laboratory Donning and Doffing Personal Protective Equipment (PPE) Checklist for Viral Hemorrhagic Fevers (VHFs)*](#_Example_Laboratory_Donning) - **Trained Observer** should assist and guide PPE donning using the checklist | |
| **🞎** | Lab Tech | Place a double-lined biohazard waste tub near the BSC to receive waste | |
| **🞎** | Lab Tech | Don third pair of nitrile examination gloves | |
| **🞎** | Lab Tech | Discard all sealed, bagged waste by gently placing into the double-lined biohazard waste tub | |
| **🞎** | Lab Tech | Replace plastic cover on top of biohazard waste tub | |
| **🞎** | Lab Tech | Perform hand hygiene on outer gloves using facility approved disinfectant wipe or alcohol-based hand rub | |
| **🞎** | Lab Tech, Observer | Doff PPE according to the [*Laboratory Donning and Doffing Personal Protective Equipment (PPE) Checklist for Viral Hemorrhagic Fevers (VHFs)*](#_Example_Laboratory_Donning) - **Trained Observer** should assist and guide PPE donning using the checklist | |

## Preparation of Biohazard Waste Tub for Daily Environmental Services Pickup

| 🗹 | **Personnel** | **Step** | |
| --- | --- | --- | --- |
| **🞎** | Observer | Record the following information:  **Lab Tech:** ______________________  **Date:** __________ | **Observer:** ______________________  **Time:** __________ |
| **🞎** | Team Member | At the end of each day of testing (or sooner if >1/2 full), a team member will contact Environmental Services (pager or phone #) for waste pickup | |
| **🞎** | Lab Tech, Observer | **Lab Tech** should don PPE according to the [*Laboratory Donning and Doffing Personal Protective Equipment (PPE) Checklist for Viral Hemorrhagic Fevers (VHFs)*](#_Example_Laboratory_Donning) - **Trained Observer** should assist and guide PPE donning using the checklist | |
| **🞎** | Lab Tech | Place one unlined biohazard waste tub and one single-lined biohazard waste tub near door of laboratory | |
| **🞎** | Lab Tech | Don a third pair of single use nitrile gloves | |
| **🞎** | Lab Tech | Pour a one-step, quaternary-based disinfectant cleaner (e.g., Virex Plus) into the used biohazard waste bag to sufficiently cover the surface of materials in the bag | |
| **🞎** | Lab Tech | Tie the bag by gathering the corners of the bag, twisting them into a long “neck” and then tying the bag using an overhand knot – the bag should be tied closed with a minimum of entrapped air | |
| **🞎** | Lab Tech | Lift the bag out of the tub and wipe the outside of the bag with a facility-approved disinfectant wipe | |
| **🞎** | Lab Tech | Place bag in lined biohazard waste tub near laboratory door with the tied knot facing up | |
| **🞎** | Lab Tech | Tie the biohazard waste tub bag around the sealed bag by gathering the corners of the outer bag, twisting them into a long “neck” and then tying using an overhand knot – the bag should be tied closed with a minimum of entrapped air. | |
| **🞎** | Lab Tech | Lift the bag out of the tub and wipe the outside of the bag with a facility-approved disinfectant wipe | |
| **🞎** | Lab Tech | Place bag in unlined biohazard waste tub near laboratory door with the tied knot facing up | |
| **🞎** | Lab Tech | Perform hand hygiene on gloves using alcohol-based hand rub | |
| **🞎** | Lab Tech | Doff PPE using [*Laboratory Donning and Doffing Personal Protective Equipment (PPE) Checklist for Viral Hemorrhagic Fevers (VHFs)*](#_Example_Laboratory_Donning) as a guide - make sure PPE is discarded into a second double bagged cardboard medical waste box or bin | |

## Environmental Services Daily Waste Pickup

| 🗹 | **Personnel** | **Step** | |
| --- | --- | --- | --- |
| **🞎** | Observer | Record the following information:  **Waste Handler:** ____________________  **Observer:** ____________________ | **Lab Tech:** ____________________  **Date:** __________ **Time:** __________ |
| **🞎** | Lab Tech | Don disposable lab coat and single use nitrile examination gloves | |
| **🞎** | Hazardous Waste Worker, Environmental Services Manager | An **Environmental Services Hazardous Waste Worker**, accompanied by an **Environmental Services Manager**, will bring a clean cart and 55-gallon cardboard (or alternative) drum provided by the facility’s biohazard waste removal vendor lined with 6-mil polyethylene liner and adsorbent material to the location and place the drum on the floor in the clean corridor at the entrance to the lab | |
| **🞎** | Lab Tech, Hazardous Waste Worker | Working together as needed, the **Environmental Services Hazardous Waste Worker** (in the clean corridor outside the Lab) and the **Lab Tech** (at the door to the clean corridor) will place the disinfected, double-bagged waste into the open drum  ***Note:*** Each drum will accommodate 2 bags | |
| **🞎** | Lab Tech | Perform hand hygiene on gloves using alcohol-based hand rub | |
| **🞎** | Lab Tech | Discard PPE (disposable lab coat and gloves) into open drum | |
| **🞎** | Lab Tech | Perform hand hygiene on bare hands using alcohol-based hand rub | |
