# Supplementary material for: Enabling laboratory readiness and preparedness for the evaluation of suspected viral hemorrhagic fevers: development of a laboratory toolkit
Source: Infect Control Hosp Epidemiol. 2024 Oct 11;45(9):1043–9. doi: 10.1017/ice.2024.143 (PMC11518664; doi:10.1017/ice.2024.143)
Supplement: Turbett et al. supplementary material 3 — Turbett et al. supplementary material [file S0899823X24001430sup003.docx]

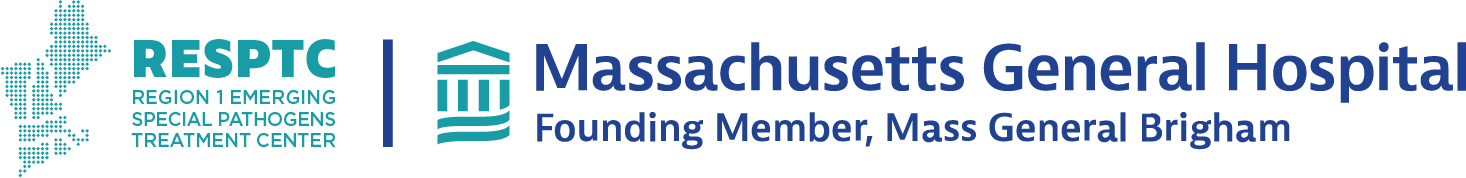


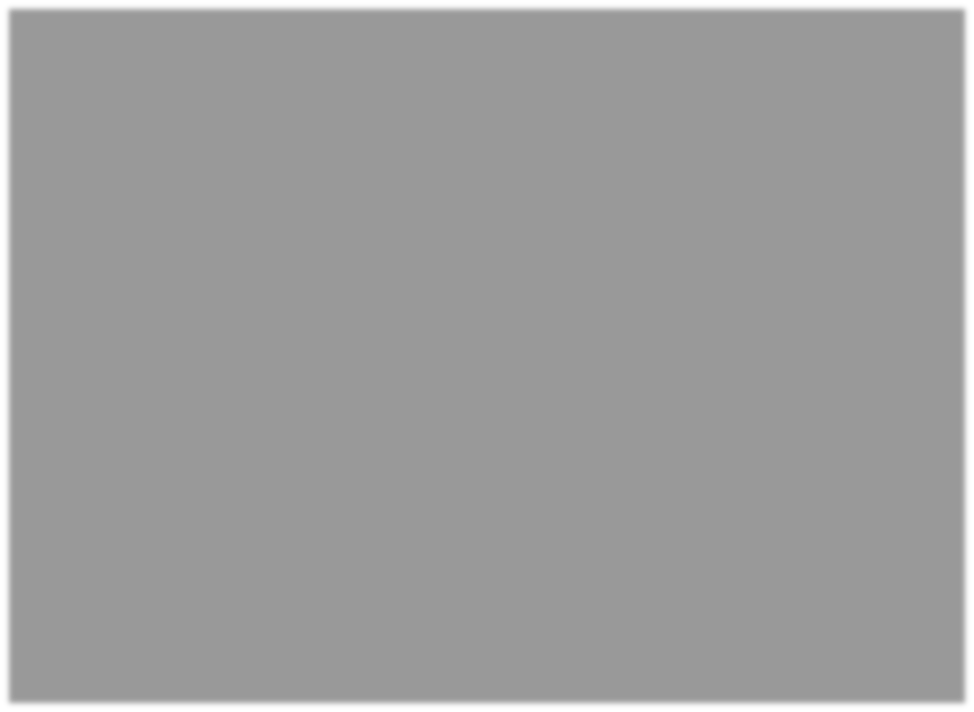

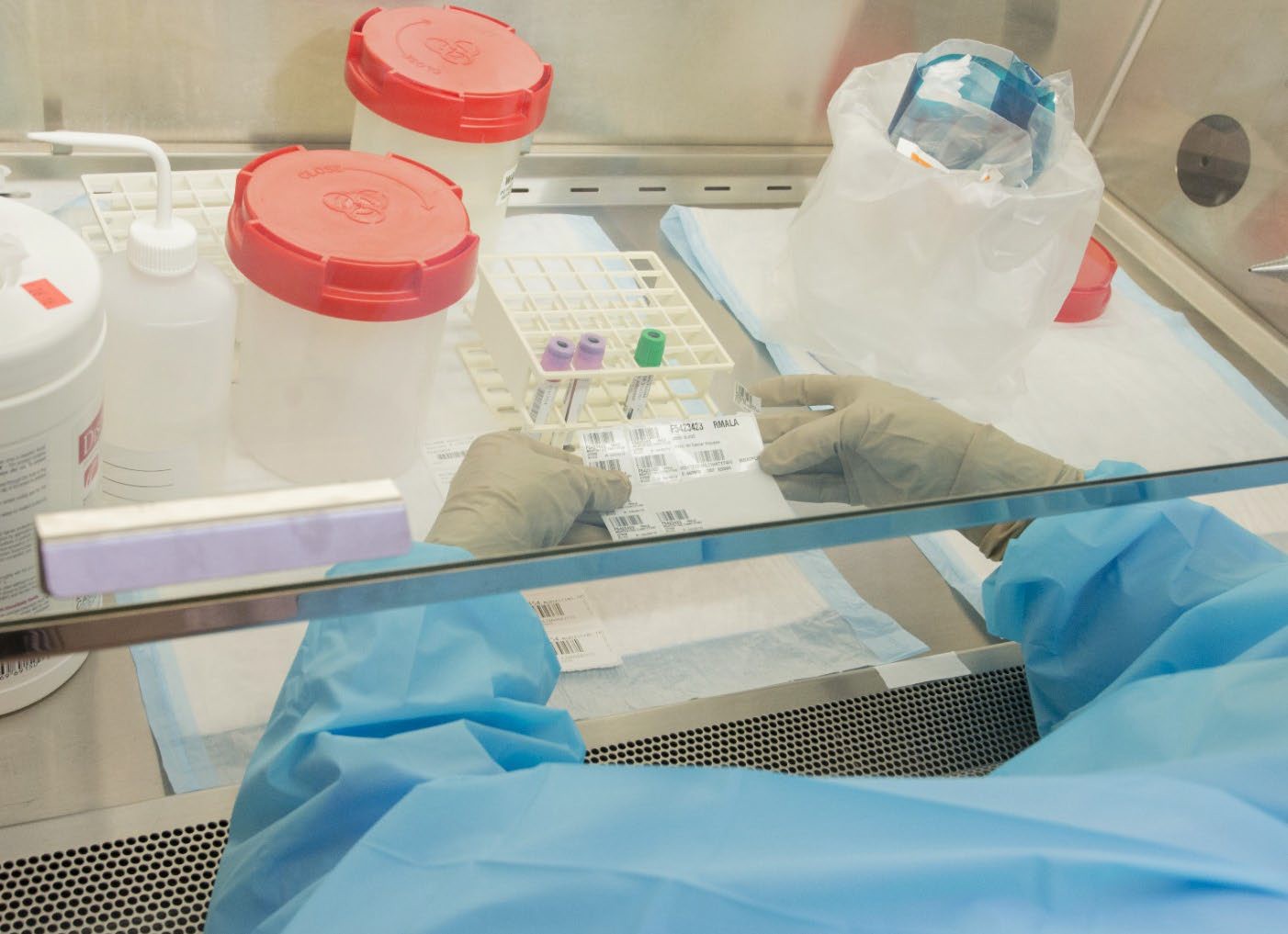


# Laboratory Testing Toolkit for a Suspect Viral Hemorrhagic Fever (VHF) Patient

*November 2023*

**1**

Table of Contents

[Introduction 3](#_bookmark0)

[How to use this document 3](#_bookmark1)

[Applicability and Scope 3](file://cifs2/mgh_bcu%24/Manuscripts/Health%20Security%20October%202023%20VHF%20Toolkit/Health%20Security%20Manuscript_VHF%20Lab%20Toolkit%20Attachments.docx#_Toc148090365)

[Rationale 3](#_bookmark2)

[Planning Assumptions 3](#_bookmark3)

[Infection Control, Waste Management, and Occupational Health 4](#_bookmark5)

[Infection Control Considerations 4](#_bookmark6)

[Cleaning and Disinfection of Equipment 5](#_bookmark8)

[Waste Management 5](#_bookmark9)

[Occupational Health 5](#_bookmark10)

[External Infection Control, Waste Management, and Occupational Health Resources 6](#_bookmark11)

[Pre-Analytic Considerations 7](#_bookmark12)

[Lab Menus 7](#_bookmark13)

[Laboratory “Go Kit” Cart 7](#_bookmark14)

[Specimen Collection Supply Kits 7](#_bookmark15)

[Specimen Transport from the Patient Bedside to Facility Lab 7](#_bookmark16)

[Specimen Storage in the Facility Lab 8](#_bookmark18)

[Specimen Accessioning in the Facility Lab 8](#_bookmark19)

[External Pre-Analytic Consideration Resources 8](#_bookmark20)

[Analytic Considerations 9](#_bookmark21)

[Required Equipment and Testing Devices 9](#_bookmark22)

[Laboratory Test Veriﬁcation, Training, Proﬁciency, and Competency 9](#_bookmark23)

[Laboratory Equipment Maintenance 9](#_bookmark24)

[Laboratory Staﬃng Assignments 9](#_bookmark25)

[Biosafety Cabinet (BSC) Preparation 9](#_bookmark26)

[Specimen Processing 10](#_bookmark27)

[Resulting 10](#_bookmark28)

[External Processing and Resulting Resources 10](#_bookmark29)

[Post-Analytic Considerations 11](#_bookmark30)

[Transport of Specimens from Facility to Public Health Laboratory 11](#_bookmark31)

[Specimen Packaging 11](#_bookmark32)

[External Post-Analytic Resources 11](#_bookmark33)

[Acronyms, Abbreviations, and Terms 12](#_bookmark34)

## Introduction

### How to use this document

This document compiles resources to support facility-level planning for initial laboratory testing of a suspect viral hemorrhagic fever (VHF) patient. Pathogens that fall into this category include those that cause Crimean-Congo Hemorrhagic Fever (CCHF), Ebola Virus Disease (EVD), Lassa Fever, and Marburg Virus Disease (MVD).

This document does not include considerations for specimen collection processes, testing occurring at the bedside, or testing for non-VHF high consequence infectious diseases (HCIDs).

**Applicability and Scope**

Guidance from your state and local health departments supersedes the information in these documents. The information included is intended to serve as a template to facilitate planning and preparedness activities related to initial laboratory testing of a suspect VHF patient.

### Rationale

Individuals who meet the criteria to have a suspect VHF will often have an alternative diagnosis. For example, travelers returning from areas currently experiencing a VHF outbreak are more likely to have febrile infections other than a VHF (e.g., malaria). Timely identification of alternative diagnoses, facilitated through access to essential laboratory testing, is necessary to provide timely and appropriate patient care. This document provides resources to assist clinical laboratories in performing initial laboratory testing necessary for the evaluation and management of a suspect VHF patient.

### Planning Assumptions

- Facilities will initiate the Identify-Isolate-Inform algorithm for a suspect VHF patient.
- Healthcare personnel (HCP) will be able to successfully don appropriate personal protective equipment (PPE) to assess and collect specimens from suspect VHF patients.
- Facilities will follow prior internally developed procedures for safe specimen collection based on Centers for Disease Control and Prevention (CDC) guidance.[^1^](#_bookmark4)
- VHF diagnostic testing will be performed at the state public health and/or CDC laboratories and will require coordination with these entities prior to specimen collection.
- All personnel who handle human specimens will comply with the [Occupational Safety and Health](https://www.osha.gov/pls/oshaweb/owadisp.show_document?p_id=10051&p_table=STANDARDS) [Administration (OSHA) Bloodborne Pathogens Standard (29 CFR § 1910.1030).](https://www.osha.gov/pls/oshaweb/owadisp.show_document?p_id=10051&p_table=STANDARDS)
- Facilities will have an established plan to manage all waste generated in the management of a patient with a suspected VHF, which is considered Category A waste, until they have been determined to no longer meet criteria.
- Specimens collected from a suspect VHF patient will be packaged and shipped as Category A infectious substances in accordance with the [Department of Transportation (DOT) Hazardous Materials](https://www.ecfr.gov/current/title-49/subtitle-B/chapter-I/subchapter-C) [Regulations (HMR) Title 49 Code of Federal Regulations (CFR) 173.196](https://www.ecfr.gov/current/title-49/subtitle-B/chapter-I/subchapter-C).

^1^ CDC, Guidance for Collection, Transport, and Submission of Specimens for Ebolavirus Testing, <https://www.cdc.gov/vhf/ebola/laboratory-personnel/specimens.html>

## Infection Control, Waste Management, and Occupational Health

### Infection Control Considerations

Your institution’s local Infection Control team must be involved in planning for handling specimens from suspect VHF patients. The Infection Control team will partner with your clinical laboratory and emergency preparedness teams to conduct a site-specific assessment to identify appropriate engineering, administrative, environmental controls, and PPE. Recommended safe work practices include:

- Ensure HCPs have been trained and evaluated in all recommended protocols related to specimen

handling and processing.

- Ensure adherence to established workplace safety programs (e.g., OSHA’s Bloodborne Pathogens, PPE, and respiratory protection standards).
- Ensure HCPs have demonstrated competency with donning and doffing appropriate PPE through testing

and assessment.

- Designate separate PPE donning and doffing spaces, when possible.
- Access to areas should be limited to essential personnel only. Log the entry and exit of all personnel who enter laboratory areas containing specimens from a patient with a suspected VHF – see the Appendix for an example [*Room Entry Log*.](#_bookmark45)
- A trained observer should monitor and coach donning and doffing, ensuring adherence to protocols.
- Clean and disinfect work areas using a facility-approved, disinfectant found on the United States Environmental Protection Agency’s (EPA) List Q (EPA Disinfectants for Emerging Viral Pathogens). An EPA List L (EPA Disinfectants for Use Against Ebola Virus) disinfectant must be used with confirmed or suspect Ebolavirus patients.

**PPE.** While a variety of PPE combinations may be considered, facilities should select and standardize PPE ensembles appropriate for HCPs via a risk assessment based on their roles and required tasks. See the Appendix for an example [*Laboratory Infection Control Task Table Guidelines for Viral Hemorrhagic Fevers (VHFs)*](#_bookmark43).

Recommended PPE ensembles for laboratory personnel include the following single use, disposable

components:

- nitrile examination gloves
- solid-front, wrap-around gown extending to mid-calf that is fluid-resistant (ANSI/AAMI BP70 Level 3) or

fluid-impermeable (ANSI/AAMI BP70 Level 4)

- surgical mask (ASTM Level 3)
- eye protection (full-face shield or goggles/safety glasses with side shield) Use of PPE beyond CDC guidance[^2^](#_bookmark7) is not recommended or addressed in this toolkit.

Before handling specimens, HCPs should be evaluated for proficiency and competency in donning and doffing procedures and all necessary duties while wearing PPE. Periodic review of donning and doffing processes with staff at regular intervals is strongly recommended. A trained observer, knowledgeable about the recommended PPE, donning and doffing procedures, and PPE disposal should monitor and coach each HCP in donning and doffing using a predetermined procedure checklist. An example [*Viral Hemorrhagic Fever (VHF) Personal Protective Equipment (PPE) Checklist*](#_bookmark44) is included in the Appendix. Review of PPE protocols should be regularly performed to ensure HCPs are consistently compliant with all procedures. *Laboratory Donning and Doffing* [*Viral Hemorrhagic Fever (VHF) Personal Protective Equipment (PPE) Checklist*](#_bookmark44) is included in the Appendix. Review of PPE protocols should be regularly performed to ensure HCPs are consistently compliant with all procedures.

^2^ CDC, Guidance for U.S. Hospitals and Clinical Laboratories on Performing Routine Diagnostic Testing for Patients with Suspected Ebola Disease, <https://www.cdc.gov/vhf/ebola/laboratory-personnel/safe-specimen-management.html#anchor_1670347424527>

### Cleaning and Disinfection of Equipment

Both the local infection control department and laboratory leadership determine what bleach germicidal disinfectant is acceptable for cleaning laboratory surfaces and equipment. A list of acceptable disinfectants can be found on the United States Environmental Protection Agency’s (EPA’s) List Q (EPA Disinfectants for Emerging Viral Pathogens). An EPA List L (EPA Disinfectants for Use Against Ebola Virus) disinfectant must be used for confirmed or suspect Ebolavirus patients. Disinfectants found on these lists meet the CDC’s criteria for disinfection of hard, non-porous surfaces.

Some EPA-approved products can be corrosive if used in high concentrations, requiring laboratories to ensure that selected disinfectants are compatible with laboratory instrumentation. Laboratories must review the manufacturers’ instrument user manuals to determine if disinfection of their laboratory instruments, including the interior surfaces, is required. Laboratory equipment, such as pipettes, should be sequestered pending confirmatory test results.

### Waste Management

Facilities may choose to sequester waste pending confirmatory testing. To do this, facilities must dedicate a secured, access-controlled room for waste sequestration. If VHF has been ruled-out, waste can be managed routinely according to the appropriate pathogen classification standards. If a VHF or other Category A Pathogen is confirmed, waste disposal should follow your facility’s Category A Waste Disposal Protocol. Consult your state Department of Environmental Protection (DEP) for state-specific considerations and additional guidance related to waste management. See the Appendix for an example [*Viral Hemorrhagic Fever (VHF) Category A Waste*](#_bookmark47) [*Handling Checklist*.](#_bookmark47)

### Occupational Health

Laboratories should establish procedures for tracking and monitoring all HCP involved with specimen management and all HCP should be instructed to immediately report any potential exposure to blood, body fluids, or other infectious materials to their local occupational health services department. Local occupational health services should have clear guidance for what constitutes a high-risk exposure and have established plans for monitoring and management of HCP who meet this definition, based on CDC guidelines, if a diagnosis of VHF is confirmed.

**External Infection Control, Waste Management, and Occupational Health Resources**

| **Resource** | **Link** | **Last Accessed** |
| --- | --- | --- |
| CDC: Ebola Disease: Laboratory Testing | [https://www.cdc.gov/vhf/ebola/lab](https://www.cdc.gov/vhf/ebola/laboratory-personnel/index.html)  [oratory-personnel/index.html](https://www.cdc.gov/vhf/ebola/laboratory-personnel/index.html) | October 2023 |
| CDC: Guidance for Malaria Diagnosis in Patients with Suspect  Ebolavirus or Marburg Virus Infection in the United States | [https://www.cdc.gov/malaria/new_](https://www.cdc.gov/malaria/new_info/2014/malaria_ebola.htm) [info/2014/malaria_ebola.htm](https://www.cdc.gov/malaria/new_info/2014/malaria_ebola.htm) | October 2023 |
| CDC: Infection Prevention and Control Recommendations for Patients in U.S. Hospital who are Suspected or Confirmed to have Selected Viral Hemorrhagic Fevers (VHF) | [https://www.cdc.gov/vhf/ebola/clin](https://www.cdc.gov/vhf/ebola/clinicians/evd/infection-control.html) [icians/evd/infection-control.html](https://www.cdc.gov/vhf/ebola/clinicians/evd/infection-control.html) | October 2023 |
| CDC: Interim Guidance for Environmental Infection Control in Hospital for Ebola Virus | [https://www.cdc.gov/vhf/ebola/clin](https://www.cdc.gov/vhf/ebola/clinicians/cleaning/hospitals.html) [icians/cleaning/hospitals.html](https://www.cdc.gov/vhf/ebola/clinicians/cleaning/hospitals.html) | October 2023 |
| CDC: Interim Guidance on Risk Assessment and Management of Persons with Potential Ebolavirus or Marburgvirus Exposure | [https://www.cdc.gov/quarantine/v](https://www.cdc.gov/quarantine/vhf/interim-guidance-risk-assessment.html) [hf/interim-guidance-risk-](https://www.cdc.gov/quarantine/vhf/interim-guidance-risk-assessment.html) [assessment.html](https://www.cdc.gov/quarantine/vhf/interim-guidance-risk-assessment.html) | October 2023 |
| EPA List L: Disinfectants for Use Against Ebola Virus | [https://www.epa.gov/pesticide-](https://www.epa.gov/pesticide-registration/list-l-disinfectants-use-against-ebola-virus) [registration/list-l-disinfectants-use-](https://www.epa.gov/pesticide-registration/list-l-disinfectants-use-against-ebola-virus) [against-ebola-virus](https://www.epa.gov/pesticide-registration/list-l-disinfectants-use-against-ebola-virus) | October 2023 |
| EPA List Q: Disinfectants for Emerging Viral Pathogens (EVPs) | [https://www.epa.gov/pesticide-](https://www.epa.gov/pesticide-registration/disinfectants-emerging-viral-pathogens-evps-list-q) [registration/disinfectants-emerging-](https://www.epa.gov/pesticide-registration/disinfectants-emerging-viral-pathogens-evps-list-q) [viral-pathogens-evps-list-q](https://www.epa.gov/pesticide-registration/disinfectants-emerging-viral-pathogens-evps-list-q) | October 2023 |

## Pre-Analytic Considerations

### Lab Menus

Laboratory testing should be limited to the minimum tests necessary for evaluation and clinical management of a suspect or confirmed VHF patient. Specifically, testing should focus on identifying life-threatening electrolyte and renal abnormalities and establishing common alternative diagnosis (e.g., malaria). Clinical evaluation for anemia, thrombocytopenia, and coagulopathy can be performed in place of laboratory testing. Below is the recommended laboratory evaluation for a suspect VHF patient:

- basic metabolic panel (i.e., sodium, potassium, glucose, creatinine)
- rapid malaria assay (e.g., BinaxNOW Point-of-Care Malaria Test)
- VHF testing (to be performed at public health laboratories)

Other CDC-recommended laboratory tests for a suspect VHF patient may also be completed at the discretion of the facility based on a local risk assessment.[^3^](#_bookmark17) An example [*Viral Hemorrhagic Fever (VHF) Laboratory Testing*](#_bookmark35) [*Menu*](#_bookmark35) is included in the Appendix. Also included is an example [*Viral Hemorrhagic Fever (VHF) Risk Clinical Lab*](#_bookmark38) [*Requisition Form*.](#_bookmark38)

### Laboratory “Go Kit” Cart

A designated VHF Laboratory “Go Kit” cart, including par levels of appropriate equipment and supplies necessary to support activation of a VHF response, can support VHF preparedness efforts. An example [*Viral Hemorrhagic*](#_bookmark36) [*Fever (VHF) Laboratory “Go Kit” Cart Packing List*](#_bookmark36) is available in the Appendix. Cart contents should be tracked by laboratory personnel to ensure adequate supply and prevent use of expired products. Tracking also allows for contents to be rotated with the general laboratory consumables to minimize expiration before use.

### Specimen Collection Supply Kits

Self-contained specimen collection kits containing all supplies needed for specimen collection, including test requisition forms and specimen containers or tubes, should be created and maintained by the laboratory and deployed as needed. An example [*Viral Hemorrhagic Fever (VHF) Specimen Collection Supply Kit Packing List*](#_bookmark37) is included in the Appendix. Kit supply tracking should be maintained as per laboratory protocols, and contents should be rotated with the general laboratory cache to minimize expiration before use.

### Specimen Transport from the Patient Bedside to Facility Lab

Protocols establishing routes of intra-facility VHF specimen transport should be created, exercised, and maintained. Protocols should provide step-by-step guidance and identify responsible parties for various stages of the process (e.g., gathering necessary supplies, specimen collection, preparing and packaging samples for transport from the point of care to the facility lab, specimen transport). The following best practices should be incorporated:

- Specimens should be sealed in a specimen container that is then disinfected with a facility-approved disinfectant.
- As per OSHA Bloodborne Pathogens Standard ([29 CFR 1910.1030](https://www.osha.gov/laws-regs/regulations/standardnumber/1910/1910.1030)), the sealed specimen containers should be packaged in a durable, leakproof secondary container.

^3^ Centers for Disease Control and Prevention (CDC). (2023, April 20). Managing and Testing Routine Clinical Specimens. Ebola Disease. <https://www.cdc.gov/vhf/ebola/prevention/handling-sewage.html>

- After placing samples in the leak-proof secondary containers, all specimens should be hand-carried to the laboratory or packaging area. Pneumatic tube or other automated transport systems should not be used.
- Bagged specimens that have been disinfected with a facility-approved disinfectant and placed in a leak- proof secondary container that has been disinfected with a facility-approved disinfectant can be handled with clean, non-gloved hands during transport pending a site-specific risk assessment.
- Specimen transport should occur using a clear, unencumbered pathway; security escort may be

beneficial for this purpose.

### Specimen Storage in the Facility Lab

Short-term storage of specimens for processing by an outside laboratory may be necessary. Specimens from a suspect VHF patient should be sequestered from other clinical laboratory specimens and stored in a labeled biosafety carrier or 95kPa shipping container in a secure location within the laboratory. Specimens should be kept at 2-8°C. Only essential personnel should have access to the specimen storage location.

### Specimen Accessioning in the Facility Lab

All specimens, including those sent to state public health laboratories, need to be accessioned into the laboratory information system (LIS) using the VHF risk clinical lab requisition form provided on the front pouch of the facility specimen cooler. See the example [*Viral Hemorrhagic Fever (VHF) Risk Clinical Lab Requisition Form*](#_bookmark38) provided in the Appendix for reference. All marked tests on the requisition form should be accessioned per laboratory protocols. Accessioning of samples to be sent to state public health laboratories for VHF testing will require additional state lab requisition and specimen submission forms. A laboratory accessioning checklist to guide laboratory personnel with this process is available; see [*Viral Hemorrhagic Fever (VHF) Pre-Analytic Cooler*](#_bookmark39) [*Receipt and Accessioning Procedural Checklist*](#_bookmark39) provided in the Appendix for reference. The laboratory accessioning checklist should also include communication and reporting expectations. Standard laboratory procedures regarding specimen identification should be followed.

**External Pre-Analytic Consideration Resources**

| **Resource** | **Link** | **Last Accessed** |
| --- | --- | --- |
| CDC: Guidance for Malaria Diagnosis in Patients with Suspect Ebolavirus or Marburgvirus Infection in the United States | [https://www.cdc.gov/malaria/new_info/2014/mala](https://www.cdc.gov/malaria/new_info/2014/malaria_ebola.htm)  [ria_ebola.htm](https://www.cdc.gov/malaria/new_info/2014/malaria_ebola.htm) | October 2023 |
| CDC: Guidance for Collection, Transport and Submission of Specimens for Ebola Virus Testing in the United States (poster) | [https://www.cdc.gov/vhf/ebola/resources/pdfs/Eb](https://www.cdc.gov/vhf/ebola/resources/pdfs/Ebola-lab-guidance-508.pdf)  [ola-lab-guidance-508.pdf](https://www.cdc.gov/vhf/ebola/resources/pdfs/Ebola-lab-guidance-508.pdf) | October 2023 |
| CDC: Interim Guidance for Environmental Infection Control in Hospital for Ebola Virus | [https://www.cdc.gov/vhf/ebola/clinicians/cleaning](https://www.cdc.gov/vhf/ebola/clinicians/cleaning/hospitals.html)  [/hospitals.html](https://www.cdc.gov/vhf/ebola/clinicians/cleaning/hospitals.html) | October 2023 |
| CDC Malaria Diagnostic Tests | [https://www.cdc.gov/malaria/diagnosis_treat](https://www.cdc.gov/malaria/diagnosis_treatment/diagnostic_tools.html) [ment/diagnostic_tools.html](https://www.cdc.gov/malaria/diagnosis_treatment/diagnostic_tools.html) | October 2023 |

## Analytic Considerations

### Required Equipment and Testing Devices

Specimen manipulation (e.g., opening a tube, preparing an aliquot) and processing requires the use of a Class II or higher Biosafety Cabinet (BSC) (NSF/ANSI 49). Laboratory leadership should determine specific equipment used. Additional materials and testing devices required include:

- Handheld blood analyzer that can be operated within the Class II BSC (to perform basic chemistries).
- Rapid malaria testing kit.
- Additional consumables required for test performance and waste disposal (see [*Example Class II*](#_bookmark41) [*Biosafety Cabinet (BSC) Placemat for Analysis of Viral Hemorrhagic Fever (VHF) Specimens*](#_bookmark41) in the Appendix).

### Laboratory Test Veriﬁcation, Training, Proﬁciency, and Competency

Per Clinical Laboratory Improvement Amendments (CLIA), all laboratory testing performed at the lab facility (basic metabolic profile and malaria testing) must undergo test verification prior to reporting patient test results. Test verification requirements are determined by the facility laboratory leadership. Once verified, comprehensive training of laboratory personnel should be performed, and written policies and procedures should be provided. Individualized quality control plans (IQCP) should be implemented where applicable.

Proficiency testing (PT) and competency assessment of all in-facility laboratory testing should be performed and

reviewed by the facility laboratory director, per CLIA.

### Laboratory Equipment Maintenance

All laboratory equipment (e.g., BSC, blood analyzer) should be maintained per routine clinical laboratory

protocols based on the manufacturer’s specifications.

### Laboratory Staﬃng Assignments

Two staﬀ are recommended for specimen processing: a “Testing Tech” and a “Buddy Tech.” The “Testing Tech” is primarily responsible for completing testing processes within the BSC. The “Buddy Tech” is responsible for the set-up, cleaning and disinfection of the biosafety cabinet, as well as responsibilities outside of the BSC (e.g., recording testing results, time keeping). Checklists should deﬁne and outline responsibilities for each laboratory technologist. Example [*Laboratory Procedural Checklists for Viral Hemorrhagic Fevers (VHF)*](#_bookmark40) are included in the Appendix. Facilities may choose to combine the “Testing Tech” and “Buddy Tech” job responsibilities at their discretion.

### Biosafety Cabinet (BSC) Preparation

Special considerations should be made when preparing the BSC for specimen processing including clearly differentiating between “clean” and “dirty” areas. BSC function checks should be performed and documented as per laboratory protocols prior to specimen processing. A BSC setup checklist will help ensure the testing laboratory technologist has all equipment required for specimen processing. An example [*Class II Biosafety*](#_bookmark41) [*Cabinet (BSC) Placemat for Analysis of Viral Hemorrhagic Fever (VHF) Specimens*](#_bookmark41) is included in the Appendix.

### Specimen Processing

Specimens are handled following the Bloodborne Pathogen Standard. The following best practices should be

incorporated:

- If there is visible contamination on a surface at any point in the testing process, stop to decontaminate

area or surface with facility-approved disinfectant wipes before proceeding.

- Tests should be repeated if error codes are obtained; do not repeat abnormal results.

### Resulting

All results should be recorded on the laboratory test result form and provided to the clinical care team in real- time. For ease of reporting, an example [*Laboratory Test Result Form*](#_bookmark42) is included in the Appendix. Additionally, all test results should be electronically entered into the LIS. Critical results should be handled per clinical results policies.

**External Processing and Resulting Resources**

| **Resources** | **Link** | **Last Accessed** |
| --- | --- | --- |
| CDC: Guidance for U.S. Hospitals and Clinical Laboratories on Performing Routine Diagnostic Testing for Patients with Suspected Ebola Disease | [https://www.cdc.gov/vhf/ebola/laboratory](https://www.cdc.gov/vhf/ebola/laboratory-personnel/safe-specimen-management.html)  [-personnel/safe-specimen-](https://www.cdc.gov/vhf/ebola/laboratory-personnel/safe-specimen-management.html) [management.html](https://www.cdc.gov/vhf/ebola/laboratory-personnel/safe-specimen-management.html) | October 2023 |
| CDC OneLab REACH Training: Fundamentals of Working Safely in a Biological Safety Cabinet | [https://reach.cdc.gov/course/fundame](https://reach.cdc.gov/course/fundamentals-working-safely-biological-safety-cabinet) [ntals-working-safely-biological-safety-](https://reach.cdc.gov/course/fundamentals-working-safely-biological-safety-cabinet) [cabinet](https://reach.cdc.gov/course/fundamentals-working-safely-biological-safety-cabinet) | October 2023 |
| CLIA Verification of Performance Specifications | [https://www.cms.gov/regulations-and-](https://www.cms.gov/regulations-and-guidance/legislation/clia/downloads/6064bk.pdf) [guidance/legislation/clia/downloads/6](https://www.cms.gov/regulations-and-guidance/legislation/clia/downloads/6064bk.pdf) [064bk.pdf](https://www.cms.gov/regulations-and-guidance/legislation/clia/downloads/6064bk.pdf) | October 2023 |

## Post-Analytic Considerations

### Transport of Specimens from Facility to Public Health Laboratory

Inter-facility specimen transport guidance documents should be formalized in advance. These documents should consider infection control guidance, communication methods and expectations, and identify step-by-step guidance and responsible parties for specimen packaging, required shipping paperwork and package labeling, and any security considerations.

### Specimen Packaging

All specimens must be packaged and shipped in accordance with the DOT’s [Hazardous Materials Regulation](https://www.ecfr.gov/current/title-49/subtitle-B/chapter-I/subchapter-C/part-171) [(HMR) 49 CFR 171-180](https://www.ecfr.gov/current/title-49/subtitle-B/chapter-I/subchapter-C/part-171). Packaging must be completed by someone trained and certified in compliance with DOT or the International Air Transport Association (IATA) requirements every 2 years.

Specimens must be packaged using the triple packaging system. The triple packing system includes: 1) a primary container (sealable specimen container) wrapped with absorbent material; 2) a watertight, leak-proof secondary container; and 3) an outer shipping package that meets Category A shipping requirements. An example [*DOT*](#_bookmark46) [*Shipper’s Manifest Form for Division 6.2 Materials (Category A/Category B)*](#_bookmark46) is included in the Appendix.

Facility laboratory personnel should coordinate with the receiving laboratory on appropriate specimen temperature regulation conditions. Generally, specimens shipped to a state lab or other Laboratory Response Network (LRN) facility require cold packs, and specimens sent to the CDC for testing should be sent on dry ice and arrive at <-20°C.

**External Post-Analytic Resources**

| **Resources** | **Link** | **Last Accessed** |
| --- | --- | --- |
| CDC: Packaging and Shipping Clinical Specimens Diagram | [https://www.cdc.gov/vhf/ebola/labor](https://www.cdc.gov/vhf/ebola/laboratory-personnel/shipping-specimens.html) [atory-personnel/shipping-](https://www.cdc.gov/vhf/ebola/laboratory-personnel/shipping-specimens.html) [specimens.html](https://www.cdc.gov/vhf/ebola/laboratory-personnel/shipping-specimens.html) | October 2023 |
| EPA List L: Disinfectants for Use Against Ebola Virus | [https://www.epa.gov/pesticide-](https://www.epa.gov/pesticide-registration/list-l-disinfectants-use-against-ebola-virus) [registration/list-l-disinfectants-use-](https://www.epa.gov/pesticide-registration/list-l-disinfectants-use-against-ebola-virus) [against-ebola-virus](https://www.epa.gov/pesticide-registration/list-l-disinfectants-use-against-ebola-virus) | October 2023 |
| EPA List Q: Disinfectants for Emerging Viral Pathogens (EVPs) | [https://www.epa.gov/pesticide-](https://www.epa.gov/pesticide-registration/disinfectants-emerging-viral-pathogens-evps-list-q) [registration/disinfectants-emerging-](https://www.epa.gov/pesticide-registration/disinfectants-emerging-viral-pathogens-evps-list-q) [viral-pathogens-evps-list-q](https://www.epa.gov/pesticide-registration/disinfectants-emerging-viral-pathogens-evps-list-q) | October 2023 |

## Acronyms, Abbreviations, and Terms

| **AAMI** | Association for the Advancement of Medical Instrumentation |
| --- | --- |
| **ANSI** | American National Standards Institute |
| **ASTM** | American Society for Testing and Materials |
| **BSC** | Biosafety Cabinet |
| **CCHF** | Crimean-Congo Hemorrhagic Fever |
| **CDC** | U.S. Centers for Disease Control and Prevention |
| **CFR** | Code of Federal Regulations |
| **CLIA** | Clinical Laboratory Improvement Amendments |
| **DEP** | Department of Environmental Protection |
| **DOT** | U.S. Department of Transportation |
| **EPA** | U.S. Environmental Protection Agency |
| **EVD** | Ebola Virus Disease |
| **EVP** | Emerging Viral Pathogens |
| **HCID** | High Consequence Infectious Disease |
| **HCP** | Healthcare Personnel |
| **HMR** | Hazardous Materials Regulation (DOT) |
| **IATA** | International Air Transport Association |
| **IQCP** | Individualized Quality Control Plans |
| **LIS** | Laboratory Information System |
| **LRN** | Laboratory Response Network |
| **MVD** | Marburg Virus Disease |
| **NSF** | National Sanitation Foundation |
| **OSHA** | Occupational Safety and Health Administration |
| **PPE** | Personal Protective Equipment |
| **PT** | Proficiency Testing |
| **VHF** | Viral Hemorrhagic Fever |
